# Old Acquaintances and Novel Complex Structures for the Ni(II) and Cu(II) Complexes of *bis*-Chelate Oxime–Amide Ligands

**DOI:** 10.3390/molecules29020522

**Published:** 2024-01-20

**Authors:** Carla Bazzicalupi, Craig Grimmer, Igor Vasyl Nikolayenko

**Affiliations:** 1 Department of Chemistry “Ugo Schiff”, University of Florence, Via della Lastruccia 3, 50019 Sesto Fiorentino, Italy; 2 School of Chemistry and Physics, University of KwaZulu-Natal, Private Bag X01, Scottsville 3209, Pietermaritzburg 3200, South Africa; grimmerc@ukzn.ac.za (C.G.); nikolayenkoigor77@gmail.com (I.V.N.)

**Keywords:** Ni(II) and Cu(II) complexes of *bis*-chelate oxime–amide ligands, molecular and crystal structures, metal ion rods

## Abstract

In the process of systematically studying the methylhydroxyiminoethaneamide *bis*-chelate ligands with polymethylene spacers of different lengths, L1–L3, and their transition metal complexes, a number of new Ni(II) and Cu(II) species have been isolated, and their molecular and crystal structures were determined using single-crystal X-ray diffraction. In all of these compounds, the divalent metal is coordinated by the ligand donor atoms in a square-planar arrangement. In addition, a serendipitously discovered new type of neutral Ni(II) complex, where the propane spacer of ligand L2 underwent oxidation to the propene spacer, and one of the amide groups was oxidised to the ketoimine, is also reported. The resulting ligand L2′ affords the formation of neutral planar Ni(II) complexes, which are assembled in the solid state on top of each other, and yield two polymorphic structures. In both structures, the resulting infinite, exclusively parallel metal ion columns in ligand insulation may serve as precursor materials for sub-nano-conducting connectors. Overall, this paper reports the synthesis and characterisation of seven new anionic, cationic, and neutral Ni(II) and Cu(II) complexes, their crystal structures, as well as experimental and computed UV–Vis absorption spectra for two structurally similar Ni(II) complexes, yellow and red.

## 1. Introduction

**Scheme 1:**
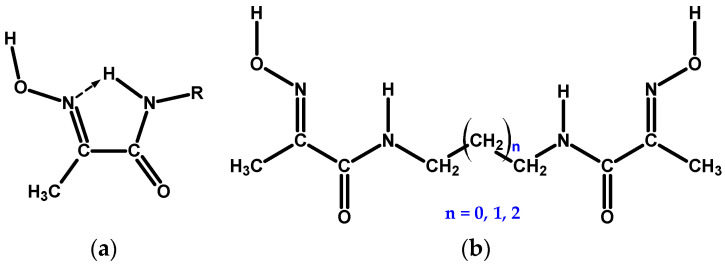
(**a**) 1-Methylhydroxyiminoethaneamide, *mhiea*, chelating moiety in free state. (**b**) *Bis*-chelate oxime–amide ligands considered in this study: *n* = 0 for L1, *n* = 1 for L2, and *n* = 2 for L3.

**Scheme 2:**
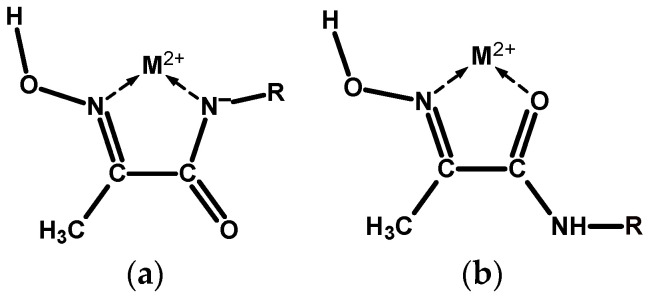
(**a**) Coordination of a divalent metal ion M^2+^ by the *mhiea* moiety in an N_ox_N_ad_ chelate mode. (**b**) Similar coordination in an N_ox_O_ad_ chelate mode.

Coordination chemistry of the hydroxyimino (oxime) group is extensive and continues to grow [1]. Adjacent oxime and amide functional groups on a ligand, Scheme 1a, form a moiety capable of chelating divalent transition and platinum group metal ions mainly in two coordination modes, which we label here as N_ox_N_ad_ and N_ox_O_ad_, due to the rotation around the –(HON=)C—C(=O)–NH– bond, Scheme 2. In addition, coordination mode N_ox_O_ox_’ is also possible, which represents binding by the donor centres of two oxime groups. Which particular mode is realised in each case is determined by a range of factors: the softness of the metal and its affinity towards the oxygen donor, medium *p*H, metal to ligand ratio, etc. In addition, intra- and inter-molecular hydrogen bonding interactions play a prominent role in the conformational states of these ligands, and in the coordination chemistry of their complexes. The ambivalent nature of the oxime group is responsible for a variety of structural types of its metal complexes: mono- and poly-nuclear, and homo- and hetero-nuclear, oligomeric, metal clusters. The former offer opportunities for supramolecular self-assembled structures [2], while the latter are particularly interesting for bioinorganic modelling of the active centres of metallo-enzymes [3,4]. Mediation by the oxime group of exchange interaction (mostly antiferromagnetic) between non-zero spin 3*d* metal centres in the clusters with a bridging motif is an essential feature of such molecules [5,6].

**Scheme 3:**
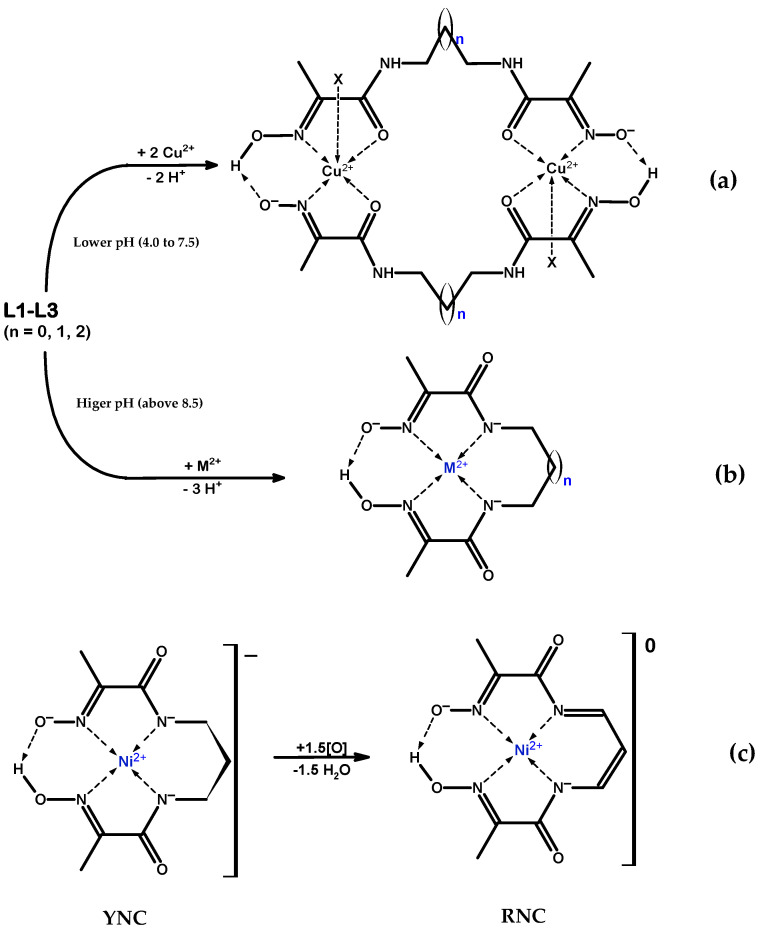
(**a**) Reaction scheme for the formation of the dimeric cationic complex core ${\left[M\left(II\right)LH_{-1}\right]}_{2}^{2+}$ with 2(N_ox_O_ad_) coordination; lower pH (4.0 to 7.5). In all reported cases, there is also coordination by a monodentate ligand X from one or both (not shown) axial directions, resulting in the square-pyramidal or distorted octahedral coordination environment on the metal. (**b**) Reaction scheme for the formation of pseudo-macrocyclic anionic complex core ${\left[M\left(II\right)LH_{-3}\right]}^{-}$ with 2(N_ox_N_ad_) coordination; higher pH (above 8.5). (**c**) Schematic representations of the oxidation of the yellow nickel complex (YNC) anionic core, ${\left[Ni\left(II\right)L2H_{-3}\right]}^{-}$, to the neutral red nickel complex (RNC),${\left[Ni\left(II\right)L2^{\prime }H_{-2}\right]}^{0}$; oxidation may occur by exposing the YNC to the air for a long time (route A), or by reacting YNC with O_2_ or H_2_O_2_ (route B).

*Bis*-chelate ligands with two oxime–amide moieties joined by a flexible link and their metal complexes are of particular interest. In this research, the nickel and copper ion complexes with *bis*-chelate ligands containing three polymethylene spacers of increasing length, Scheme 1b, are presented. Ni(II) and Cu(II) ions were chosen because they are amenable to the square-planar coordination imposed by the oxime–amide chelates; their complexes possess high thermodynamic stability and, being coloured, they are well suited for the study by UV–Vis titrations. H.-P. Lau and C.D. Gutsche were the first to report the synthesis of ligand L1, in relation to the catalytic decomposition of acetyl phosphate by its Ni(II) and Cu(II) complexes [7]. Since then, all three ligands considered in this study were prepared and characterised, including their crystal structures, L1 [7,8], L2 [1,9,10], and L3 [8,11]. A number of Ni(II) complexes [9,12,13,14] and Cu(II) complexes [9,11,12,13,14,15,16,17,18] have also been reported. All of the previously synthesised Ni(II) and Cu(II) complexes with ligands L1–L3 known to the authors are listed in Supplementary Materials Tables S1 and S2. The obtained metal complexes fall into two major structural types: (a) anionic complexes with 2(N_ox_N_ad_) square-planar coordination, Scheme 3a, where the ligand is thrice-deprotonated; and (b) dimeric cationic complexes with the 2(N_ox_O_ad_) coordination mode, where the metal ion is found in square-pyramidal or distorted octahedral geometry and the ligand is mono-deprotonated, Scheme 3b.

In the course of the systematic study of these ligands and their metal complexes, we serendipitously discovered a completely new red species obtained by the oxidation of the ${\left[Ni(II)L{2H}_{-3}\right]}^{-}$ complex, Scheme 3c. From this point forward, we will refer to this compound as the red nickel complex (RNC). The X-ray diffraction studies of two crystalline polymorphs of this compound revealed the presence in the solid state of infinite, exclusively parallel Ni ion columns, with all Ni centres being in direct contact. In this paper are reported the synthesis, characterisation, and crystal structures of five new Cu(II) coordination compounds with the *bis*-chelate oxime–amide ligands, and of two new red Ni(II) polymorphs of the oxidised L2 ligand, as shown in Table 1 and Table 2. In addition, experimental and quantum chemically computed UV–Vis absorption spectra for two structurally similar Ni(II) complexes, yellow and red, are presented.

## 2. Results

### 2.1. Synthesis and Characterisation

The reactions of complex formation are shown in Scheme 3.

#### 2.1.1. Cu(II) Complexes

The cationic (**1**) = $[\{Cu(II)L1H_{-1}{\}}^{+}(OH_{2}){]}_{2}^{2+}\cdot 2NO_{3}^{-}\cdot H_{2}O$, (**2**) = $[\{Cu\left(II\right)L3H_{-1}{\}}^{+}(OH_{2}){]}_{2}^{2+}\cdot 2BF_{4}^{-}\cdot {2H}_{2}O$, (**3**) = $[\{Cu\left(II\right)L3H_{-1}{\}}^{+}{Cl}^{-}{]}_{2}^{0}\cdot {4H}_{2}O$, (**4**) = $[\{Cu\left(II\right)L3H_{-1}{\}}^{+}{Cl}^{-}{]}_{2}^{0}\cdot {L3\cdot 2H}_{2}O$, and anionic (**5**) = ${\left[{{\left\{Li{\left({OH}_{2}\right)}_{3}\right\}}^{+}\{Cu\left(II\right)L2H_{-3}\}}^{-}\right]}^{0}\cdot H_{2}O$ Cu(II) complexes were synthesised using a general procedure by mixing a ligand solution with a solution of a Cu(II) salt. Cationic complexes (**1**–**4**) were prepared from the acidified ligand solutions, while the anionic complex (**5**) was prepared from a ligand solution deprotonated with a strong base (LiOH). The following options were tested: the Cu(II) salts (Cu(ClO_4_)_2_∙6H_2_O, Cu(OAc)_2_, Cu(NO_3_)_2_∙2½H_2_O, Cu(BF_4_)_2_∙*x*H_2_O); the strong base (LiOH, KOH); the bulky counter ions ([PPh_4_]^+^, [AsPh_4_]^+^, [PF_6_]^−^, [AsF_6_]^−^); and the solvent medium (MeOH, EtOH, H_2_O). The particulars of specific synthetic procedures and primary characterisation data for all complexes are provided in the Supplementary Materials (Synthesis S1–S5 and Figures S1–S4). From a larger number of Cu(II) complexes isolated, only five were successfully crystallised into a form suitable for single crystal X-ray diffraction (XRD), (**1**–**5**).

#### 2.1.2. Ni(II) Complexes

Several attempts have been made to synthesise cationic and neutral Ni(II) complexes, similar to the ones mentioned in [9]. Although some of these compounds have been isolated, no crystals suitable for XRD analysis were obtained. The anionic complexes were synthesised analogously to Cu(II) complexes. The variations included the following: the nature of Ni(II) salt (NiCl_2_∙6H_2_O, Ni(NO_3_)_2_∙6H_2_O, Ni(OAc)_2_∙4H_2_O, Ni(BF_4_)_2_∙6H_2_O, NiSO_4_∙6H_2_O); the nature of the strong base (LiOH, NaOH, KOH); possible addition of a bulky counter cation to facilitate crystal formation with a bulky complex anion ([PPh_4_]^+^, [AsPh_4_]^+^, [NMe_4_]^+^, [NEt_4_]^+^, [NBu_4_]^+^); and the nature of the solvent medium (MeOH, EtOH, CH_3_CN, H_2_O, and mixed water–alcohol solvents). Despite there being more Ni(II) complexes isolated, only crystal structures identical to those reported in [8,13] were obtained. Notwithstanding the fact that crystal structures of two polymorphs for complex (**6**) were published earlier [14], the characterisation of this compound in the original paper was limited to microanalysis and ESI-MS. In view of the representative nature of this anionic Ni(II) complex and its role in the synthesis of Ni(II)-L2′ species, we report the synthetic procedure for (**6**) = ${\left[{PPh}_{4}\right]}^{+}\cdot {\left[Ni\left(II\right)L2H_{-3}\right]}^{-}\cdot H_{2}O$ and comprehensive characterisation data for it in Supplementary Materials Synthesis S6. Notably, the yield from our procedure was much better than that in [14] (97% vs. 58%). From this point forward, we will refer to this compound as the yellow nickel complex (YNC).

#### 2.1.3. Novel Ni(II)-L2′ Red Nickel Complex (RNC)

Several attempts have been made to develop a procedure for RNC preparation in a controlled manner, starting from (a) primary ingredients, i.e., L2 and Ni(II) salt or from (b) previously isolated YNC, (**6**), and using a variety of oxidising agents, such as $O_{2}(g)$, $H_{2}O_{2}(aq)$, ${Br}_{2}(l)$, and $I_{2}(s)$, in MeOH or EtOH medium, Scheme 3c. Both routes led to RNC formation. Of the four oxidising agents tested, only the first two produced the desired outcome, with the hydrogen peroxide solution being much more effective than the dioxygen gas. Either of the alcohols could be used as a solvent, with no discernible preference between them. It appears that the use of ${{Ni(NO}_{3})}_{2}$ in route A is essential, which implies that the nitrate ion plays the role of a catalyst. An account of two synthetic procedures that afforded successful preparation of neutral red Ni complex is shown in Supplementary Materials Synthesis S7 and S8.

Polymorphs (**7**) and (**8**) both represent $\left[Ni\left(II\right)L2^{\prime }H_{-2}\right]$**^0^**, co-crystallised from the reaction mixture, and individual crystals were picked by hand for the diffraction and other analyses, Supplementary Materials Figure S19–S28.

**Route A. Yield:** No determination of RNC yield was attempted, but an estimate of about 40 percent could be made based on the relative intensity of NMR signals in the reaction mixture.

**Route B. Yield:** Again, no determination of actual RNC yield was attempted, but a conservative estimate of between 21 and 25 percent could be made, based on the NMR data.

### 2.2. X-ray Diffraction

**Figure 1:**
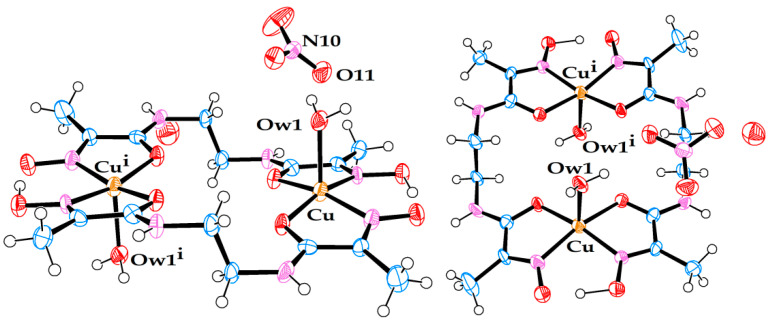
Molecular structure of dimeric cationic Cu(II) complex (**1**). (**Left**) side view. (**Right**) front view. Free nitrate ion and the third (non-coordinated) water molecule are also present in the crystal structure.

**Figure 2:**
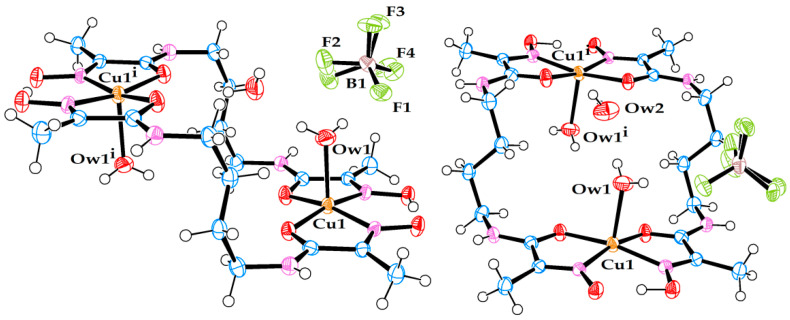
Molecular structure of dimeric cationic Cu(II) complex (**2**). (**Left**) side view. (**Right**) front view. Free tetrafluoroborate ion and the third (non-coordinated) water molecule are also present in the crystal structure. Note that the position of tetrafluoroborate anion in the crystal structure is affected by rotational disorder.

**Figure 3:**
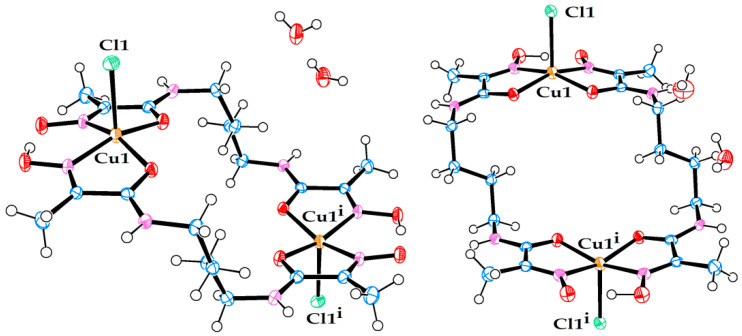
Molecular structure of dimeric cationic Cu(II) complex (**3**). (**Left**) side view. (**Right**) front view. Two non-coordinated water molecules are also present in the crystal structure.

**Figure 4:**
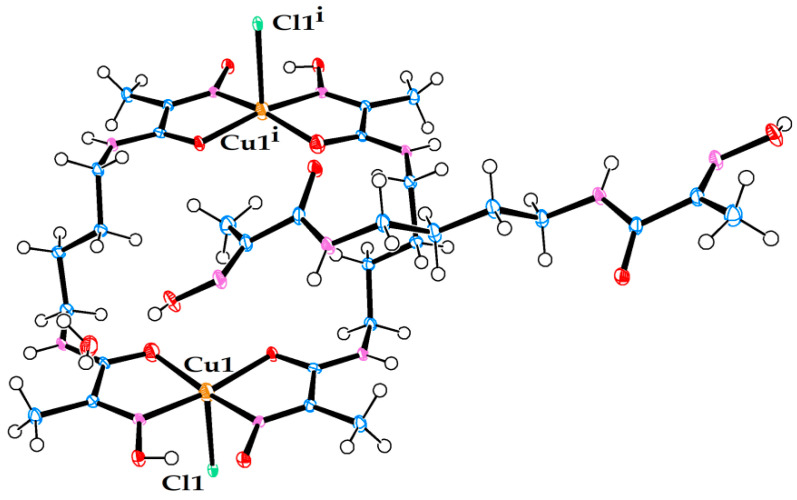
Molecular structure of dimeric cationic Cu(II) complex (**4**). A free ligand molecule and a non-coordinated water molecule are also present in the crystal structure.

**Figure 5:**
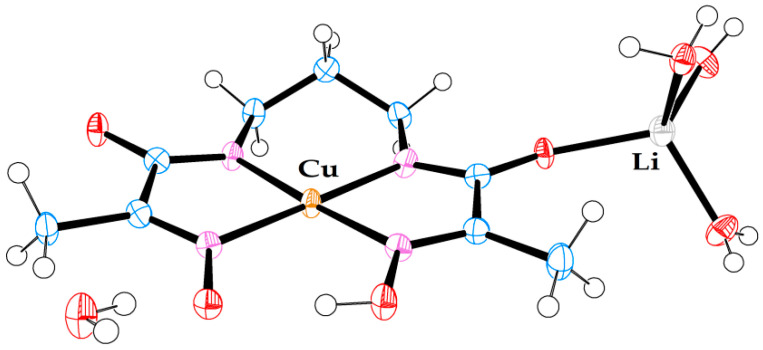
Molecular structure of anionic Cu(II) complex (**5**). A triply hydrated Li-cation is coordinated to the amide oxygen. A non-coordinated water molecule is also present in the crystal structure.

**Figure 6:**
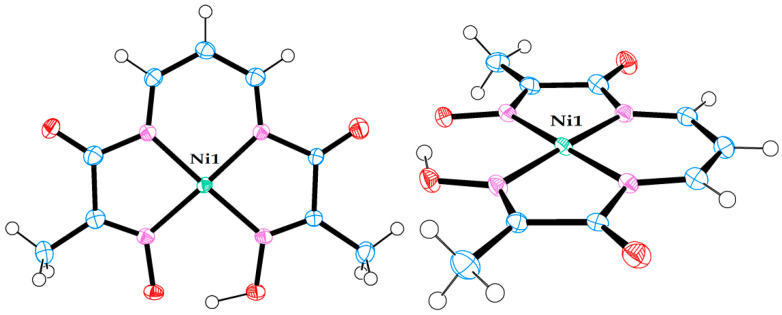
Molecular structure of neutral Ni(II) complex in crystal structure (**7**). (**Left**) view from above. (**Right**) side view.

**Figure 7:**
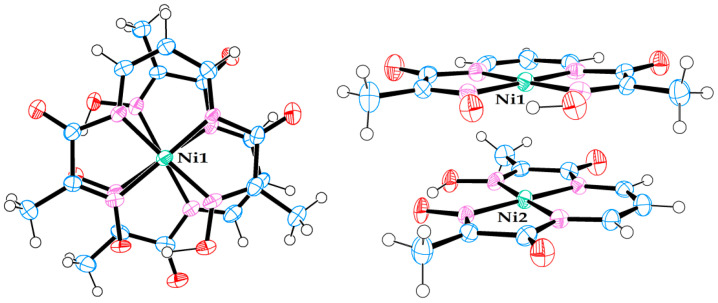
Molecular structure of neutral Ni(II) complex pair in crystal structure (**8**). (**Left**) view from above. (**Right**) side view.

A summary of the data and parameters for the single crystal diffraction experiments on metal complexes, which led to successful refinement of molecular and crystal structures, is presented in Table 3. The molecular structures of the seven metal complexes are shown in Figure 1, Figure 2, Figure 3, Figure 4, Figure 5, Figure 6 and Figure 7. In these figures, the ORTEP3 colour scheme for atoms is used: carbon—slate blue; hydrogen—white; nitrogen—orchid; oxygen—red; boron—pink; fluorine—yellow-green; chlorine—aquamarine; lithium—grey75; nickel—medium aquamarine; copper—cool copper. Selected values of bond lengths and angles for these compounds are shown in Table 4, Table 5 and Table 6.

**Figure 8:**
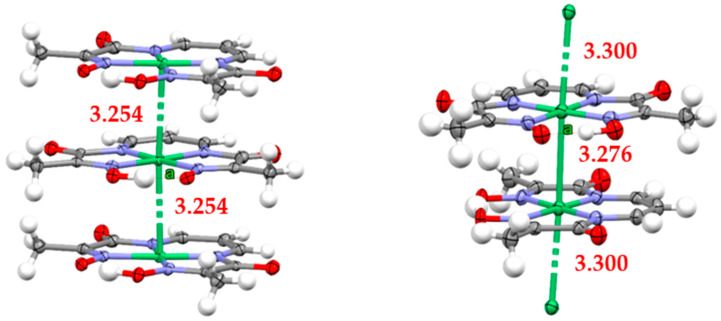
Poker-chip-type stacks of neutral complex molecules in the crystal structure of RNC viewed from the side. **Left**: (**7**); **right**: (**8**). Ni–Ni distances are stated in Å. Letter “a” indicates the site of oximato-oxime hydrogen bond.

**Figure 9:**
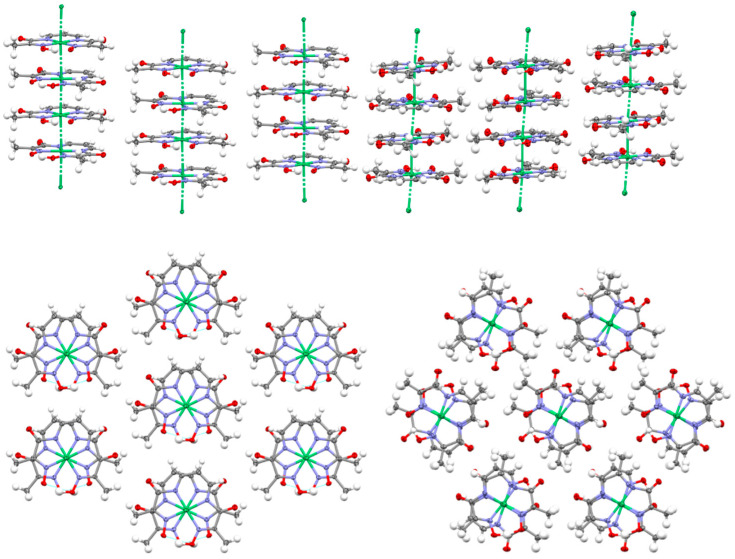
Columns of stacked neutral Ni(II) complexes in the crystal structures (**7**) (**left**) and (**8**) (**right**). (**Top**) view from the side. (**Bottom**) view from above.

The stacking of neutral RNC molecules into infinite columns is shown in Figure 8 and Figure 9.

### 2.3. Quantum Chemical Modelling

**Scheme 4:**
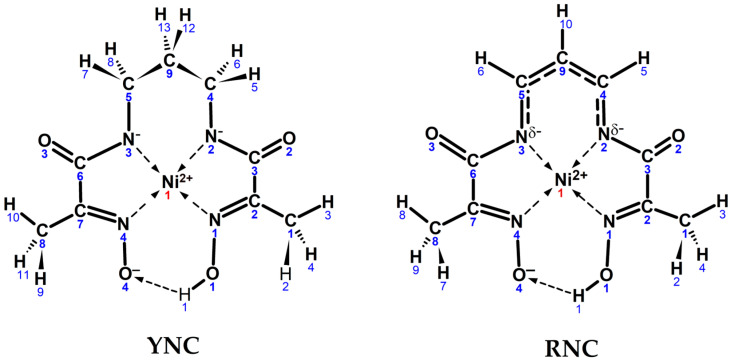
Input structures of YNC and RNC cores with the numbering schemes and realistic conformational shapes.

Quantum chemical modelling of two Ni(II) complex cores—the yellow nickel complex, ${\left[Ni\left(II\right)L2H_{-3}\right]}^{-}$, and the red nickel complex, ${\left[Ni\left(II\right)L2^{\prime }H_{-2}\right]}^{0}$—was attempted with two objectives in mind: (a) to test whether their geometry can be accurately reproduced, and (b) to account for their colours in solution (absorption spectra), as well as provide theoretical interpretation to the above. The schematic representation of two input core structures with atom numbering is shown in Scheme 4.

**Figure 10:**
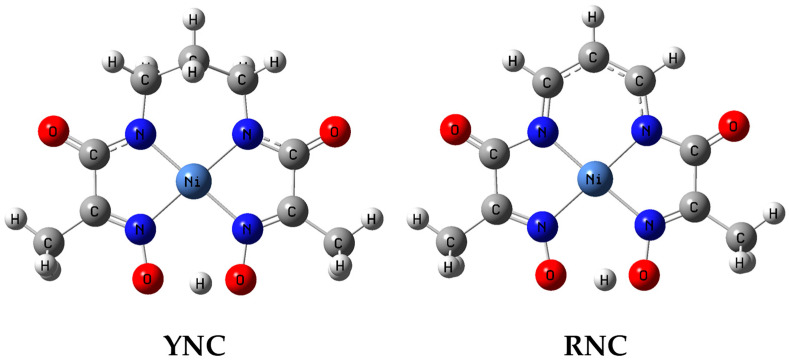
Computed geometries of the YNC and RNC cores in MeOH medium.

The same core structures, computed at the RB3LYP 6-311++G (3d, 2p) level of density functional theory in MeOH medium, are shown in Figure 10.

Representative geometric parameters of the computed structures are shown in Table 7.

**Figure 11:**
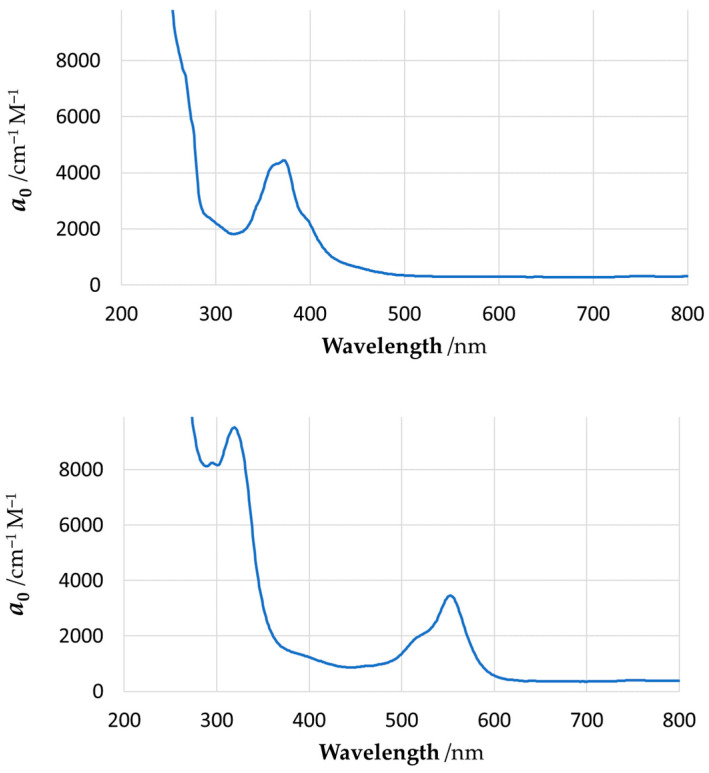
Experimental UV–Vis absorption spectra for YNC (**top**) and RNC (**bottom**) in methanolic solution. The vertical axis represents molar absorption coefficient, ***a*_0_**.

**Figure 12:**
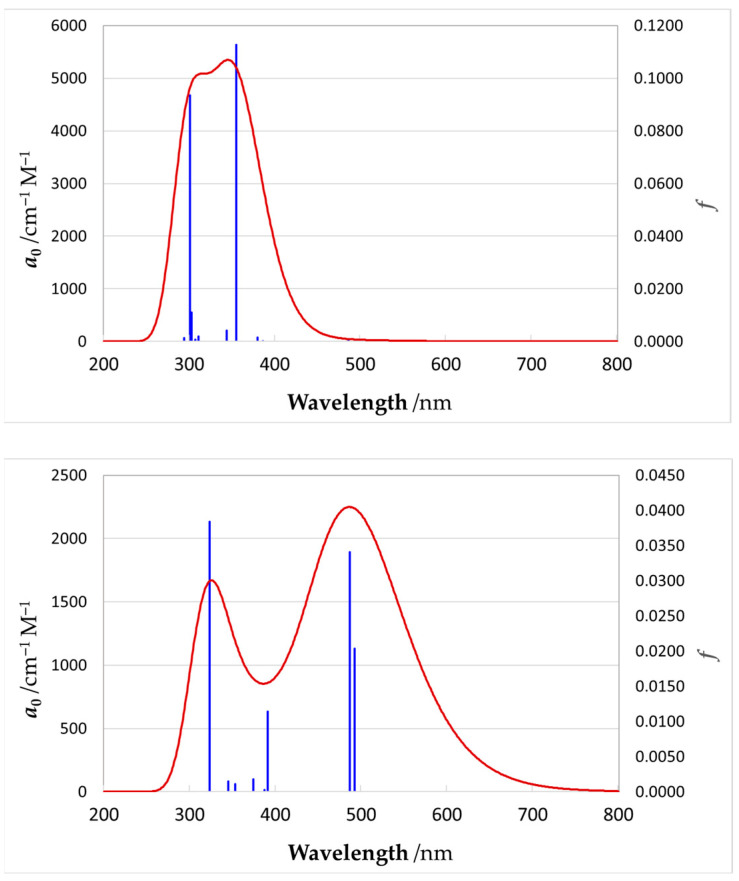
QM-computed electronic absorption spectra for YNC (**top**) and RNC (**bottom**). The left vertical axis refers to molar absorption coefficient, ***a*_0_**, while the right vertical axis shows the oscillator strength, ***f***. Vertical spectral lines represent individual adiabatic electron transitions, while the curve reflects realistically widened Gaussian profile bands.

The experimental UV–Vis unit spectra for these two complexes are shown in Figure 11, and the computed spectra are shown in Figure 12. The computed parameters of spectral lines are shown in Supplementary Materials Tables S4 and S6, and the computed parameters of the NBOs are presented in Tables S5 and S7. The strong absorption bands observed in the UV region of experimental spectra represent transitions within the ligand scaffolds; since they lie far away from the visible range, these high-energy transitions were not included in the present quantum mechanical simulation. The shapes of the computed NBOs for the YNC and RNC are shown in Supplementary Materials Figure S29, while the orbital energy diagrams are shown in Figure S30.

## 3. Discussion

In the course of preparing complexes with the ${\left[Ni\left(II\right)L2H_{-3}\right]}^{-}$ core, we serendipituously discovered that after long storage time in air, the yellow nickel complex, YNC, was converted into a neutral red nickel complex, RNC. This stirred considerable interest, as no red nickel complexes with the oxime–amide ligands had been reported at that time. It was assumed that either the Ni(II) ion in the complex underwent oxidation to the Ni(III) state, or that the ligand scaffold itself was oxidised, most likely in the propane bridge area. As it turned out, both hypotheses were realised in practice. The overall process of oxidation can be depicted, as shown in Scheme 3c. Specific measurements to elucidate the mechanism of RNC formation have not been attempted; however, from the nature of the intermediate products (identified by the NMR spectra of reaction mixture at different times), we may infer that the process of oxidation proceeds through the oxidation of Ni(II) ion in the YNC core to Ni(III), followed by stepwise intramolecular oxidation of one amide group and each of the three methylene groups of the spacer, with reduction of the Ni(III) centre back to the Ni(II) state after each step. The colour of the products obtained under similar circumstances with ligands L1 and L3 indicates that analogous processes may occur for their Ni(II) complexes too, but crystals suitable for SC-XRD were obtained using L2 only as the starting material.

Overall, this research reports the isolation in monocrystalline form of five novel Cu(II) complexes, and of two novel red Ni(II) polymorphs. XRD results for these materials complement the picture derived from literature data. The binding mode 2(N_ox_O_ad_) observed in these structures has already been reported for Cu(II) complexes with all three ligands, with the following CSD codes: KAGGUW, KAGHAD, KAGHEH, KAGHIL, NUFSUC, and NUFSUC01. In fact, only this coordination mode was reported for the complexes of ligands L1 and L3, while the formation of anionic complexes with 2(N_ox_N_ad_) coordination dominates for ligand L2, with the following CSD codes: NOXBIL, XANBET, BICGEA, XAPHUS, XEQBIF, and DUCNIZ. In the following paragraphs, a systematic discussion of the structural features of our cationic, anionic, and neutral complexes is presented.

### 3.1. Cationic Complexes

The molecular structures of dark green crystals (**1**)–(**4**) are examples of *bis*-chelate dinuclear cationic Cu(II) complexes of the *sp-tpp* type. Firstly, one needs to note that no such cationic complexes have been reported for the Ni(II) ion on account of its much lower affinity towards oxygen donors, in comparison to that of the Cu(II) ion. Secondly, all four complexes are symmetrical, with two coordination platforms being identical and parallel (Figure 1, Figure 2, Figure 3 and Figure 4). For complexes (**1**) and (**2**), axially coordinated water molecules are “inward” directed, while for complexes (**3**) and (**4**), two axially coordinated chloride anions are “outward” directed. Such axial coordination causes puckering of the complex basal plane in the direction of the axial ligand. The Cu centre distance from a basal coordination plane drawn through four donor atoms can be used to evaluate the distortions of the coordination platforms, which range from mild to severe in crystal structures (**1**)–(**4**), Table 4.

As can be seen from Figure 1, Figure 2, Figure 3 and Figure 4 and Table 4, complexes (**1**) and (**3**) are more puckered than complexes (**2**) and (**4**). Primarily, this is a result of crystal packing. Particularly interesting is the difference between the last three structures. Thus, in the most distorted structure (**3**), there is a cavity in the axial direction from the Cu centre opposite to the chloride ion, where the methyl group of the neighbouring complex is located; meanwhile, in both structures (**2**) and (**4**), there is meaningful coordination interaction of the copper ion with a fragment of the neighbouring molecule, which reduces the distortion. In the case of (**4**), there is copper coordination to the oxime oxygen donor of the free ligand. The case of (**1**) is similar to that of (**2**), with the difference of a shorter ethane spacer imposing more steric distortion on the structure.

The metal coordination environment does not appear to be influenced much by the length of the polymethylene spacer, with the bond lengths and angles being quite similar, as shown in Table 4. The obvious and most significant difference is the length of the axial bonds of the monodentate ligands coordinated to the copper ion. For the coordinated water molecules in complexes (**1**) and (**2**), these bonds are much shorter than for the chloride anions in complexes (**3**) and (**4**), 2.194(5) Å and 2.175(3) Å vs. 2.4624(8) Å and 2.5678(8) Å, respectively.

### 3.2. Anionic Complexes

Crystal structure (**5**) belongs to the class of pseudo-macrocyclic anionic complexes, numerous examples of which have been mentioned above, with one exception—a hydrated Li cation coordinated to one of the amide oxygens. Only one other example of a similar kind has been reported previously for an Ni(II) complex with ligand L1: ${\left[{\left\{{Li\left(OH_{2}\right)}_{2}\right\}}^{+}{\cdot \{Ni\left(II\right)L1H_{-3}\}}^{-}\right]}_{2}^{0}\cdot H_{2}O$ [8], CSD code JOCJUG. It is appropriate to discuss the two complexes with the coordinated Li ions in comparison to each other. The principal structural difference between the two is that in JOCJUG, two Ni complexes are bridged by a pair of Li ions joined by two water molecules in a diamond pattern, while in (**5**), the Li cation is triply hydrated and linked only to one Cu complex.

The geometric parameters of complex (**5**), Table 5, are representative of such systems. Its asymmetric nature is caused by two factors, the oximato–oxime hydrogen bond, and coordination of the Li cation to one of the amide termini.

The Cu–N_ad_ distances are shorter than Cu–N_ox_ distances, which is likely to be a reflection of the anionic nature of N_ad_ donors in these complexes. On average, the Cu–N bonds in (**5**) are longer than the corresponding Ni–N bonds in JOCJUG, which primarily can be attributed to the larger cavity size formed by ligand L2 than by ligand L1, as the square-planar coordination environment of the Cu(II) ion radius is only marginally larger (by 0.02 Å) than that of the Ni(II) ion [19]. At 102.3°, the N1–Ni–N4 angle in JOCJUG is a reflection of the wider oximato–oxime mouth, which, in turn, is caused by significant steric strain caused by a shorter ethane spacer. At 96.5°, the same angle in (**5**) is smaller, and reflects the strain relieved by the introduction of a propane spacer. Subsequently, the N2–Cu–N3 angle of 99.2° in (**5**) is much larger than the N2–Ni–N3 angle of 87.9° in JOCJUG. The oximato–oxime hydrogen bond O4⋯H1 (1.835 Å vs 1.752 Å) as well as the O4⋯O1 distance (2.633 Å vs. 2.580 Å) is shorter in (**5**) than in JOCJUG for the reasons of bridge strain discussed above. Unlike in JOCJUG, where both amide C–N bonds are nearly identical (1.317 Å vs. 1.316 Å), in (**5**), the C6–N3 bond at 1.296(4) Å is noticeably shorter than the C3–N2 bond at 1.325(4) Å. This must be a reflection of a strong coordinated pull of the sole triply hydrated Li ion in comparison to that of the quadruply hydrated dilithium bridge between the two Ni complexes in JOCJUG.

Finally, both the JOCJUG and (**5**) structures are characterised by some degree of planarity, with the exception of methyl groups and the propane bridge in (**5**), which have a predictable “flap of the envelope” conformation. Judged by the maximum deviation of an atom from an average plane drawn through 14 ligand atoms, complex JOCJUG is highly planar (0.046 Å for O4 oximato oxygen), while complex (**5**) shows noticeable curvature, from left to right, when seen from the oximato–oxime mouth (0.151 Å for C6 carbonyl carbon).

### 3.3. Neutral Complexes

Regrettably, crystallisation of new Ni(II) complexes with ligands L1–L3 was unsuccessful. However, we succeeded in the preparation and isolation of crystals of a red compound, the Ni(II) complex of an oxidised derivative of ligand L2, as shown in Scheme 3c. Our compound, ${\left\{{\left[Ni\left(II\right)L2^{\prime }H_{-2}\right]}^{0}\right\}}_{n}$, has been crystallised out in two polymorphic forms, (**7**) and (**8**). The counterions balancing the negative charge of the complex core, which are normally expected in the case of 2(N_ox_N_ad_) coordination, are conspicuously absent in these polymorphs, indicating that the Ni(II) complex is neutral and the ligand scaffold is doubly deprotonated. This conclusion was confirmed by high-resolution MS–ToF analysis. Such coordination is possible only for an oxidised derivative of ligand L2, namely for the methylhydroxyiminoethaneamide_propene_methylhydroxyiminoethaneketoimine ligand, L2′.

Geometrically, the RNC structure closely resembles that of the YNC core [14] with CSD code XAPHAY, with a few exceptions. Both molecules exhibit a fairly symmetrical metal environment, although in the RNC it is slightly more distorted than in the YNC. Thus, in the YNC core, the Ni–N_ad_ bonds are shorter than the Ni–N_ox_ bonds by approximately 0.01 Å, while in the RNC structure, this difference is approximately 0.02 Å. Also, in the YNC, the N1–Ni–N4 and N2–Ni–N3 angles are virtually identical at 96.7° and 96.6°, respectively, while in the RNC they differ more at 98.2° and 94.5°, respectively. This small asymmetry is introduced, on the one hand, by the oximato–oxime hydrogen bonded termini, and on the other hand, by incomplete delocalisation of the double bond in the propene bridge.

Major differences between the molecular structures of the YNC and RNC are observed in the area of the spacer. Thus, in comparing the RNC to the YNC, the C3–N2 and C6–N3 bonds are elongated by approximately 0.1 Å, while the N2–C4 and N3–C5 bonds are shortened by approximately 0.15 Å. The C4–C9 and C5–C9 bonds of the spacer are considerably shorter (by approximately 0.13 Å) than a typical aliphatic single C–C bond, while their proximity across structures (**7**)–(**8**) indicates a substantial degree of π-electron delocalisation in this system. Notably, the Ni complex in the two structures is entirely flat, and the bond lengths and angles related to the three bridge carbons are consistent with a delocalised double bond of the propene spacer, as shown in Table 6.

An essential feature of RNC crystal structures (**7**) and (**8**) is infinite parallel columns of planar complex molecules stacked in a manner resembling poker chips and characterised by short Ni–Ni distances. The latter form infinite parallel metal ion rods in ligand insulation—exciting precursor materials for sub-nano-conducting connectors. To the best of our knowledge, RNC crystal structures (**7**)–(**8**) are only the third example of stacked Ni(II) complexes that form infinite parallel metal ion columns without bridging atoms between metal centres. The first two are the structures based on *vic*-dioximes [20], predominantly dimethyl [21,22,23,24,25] and diphenyl [26,27,28] glyoximes, and the 3-(hydroxyamino)-3-methyl-2-butanone oximato^2−^ ligand [29]. All such structures are characterised by parallel Ni ion columns with a single metal–metal distance in the range 3.183 Å to 3.254 Å for Ni(*dmg*)_2_ (dimethylglyoxime), according to different authors, and six nearest-neighbour columns around the central stack. The Ni(*dpg*)_2_ (diphenylglyoxime) structure is also characterised by the single Ni–Ni distance of 3.547 Å, but with only four nearest neighbour columns. The channels in the latter can accommodate columns of Br or I atoms, leading to the charge transfer inclusion type complexes, where the Ni–Ni distance is lowered to 3.271(1) Å at ambient temperature [27], or to 3.223(2) Å at −160 °C [29]. A recent review of Ni complexes that form 1D stacks in which metal centres are directly aligned because of metallophilic interactions is provided in [30].

The difference in our case is that a single ligand provides the scaffold for the Ni(II) complex. The arrangement of neighbouring molecules in the stack is shown in Figure 8, while the geometry of a few neighbouring stacks in the crystal structures of (**7**) and (**8**) are shown in Figure 9. Polymorphs (**7**) and (**8**) differ in the turn angle of neighbouring complexes in a stack, 60.7° and 7.1°, respectively, yielding what can be called staggered for conformation (**7**) or almost eclipsed for (**8**). As a result, perfectly overlaid complexes in (**7**) show upright columns, with the Ni ion rods characterised by a single Ni−Ni distance of 3.254(4) Å. In the case of (**8**), the eclipsed complexes yield slightly leaning zig-zag columns (the Ni–Ni bonds are tilted alternatively by approximately 2.0° and 7.7° with respect to the normal of each coordination plane, Figure 9, top right) characterised by two alternating Ni−Ni distances of 3.276(7) Å and 3.300(7) Å. However, these small differences do not significantly alter the overall packing in these two structures. A remarkable feature of both solids is parallel growth of columns in a diamond-shaped pattern. The edge and the smaller diagonal of the lozenge (Ni⋯Ni separation) measure 10.9 Å and 9.6 Å for (**7**), and 10.3 Å and 9.1 Å for (**8**), respectively. Based on the structural evidence, it is reasonable to expect highly anisotropic behaviour, with the physical properties in the direction of Ni ion rods significantly different from the properties in orthogonal directions.

### 3.4. Computational Results

As far as the computational results are concerned, it is reasonable to consider geometric parameters of the optimised complex structures first on their own rather than in relation to the solid-state structures, as they are devoid of distortions introduced by the crystal lattice forces. Analysis of the computed geometric parameters permits drawing the following conclusions. In both complexes, the Ni(II) ion is found in a nearly perfect square-planar coordination environment. With the exception of the propane bridge in the YNC, which has a “flap of an envelope” conformation and protrudes above the plane, the rest of the ligand atoms lie very close to the average complex plane. There is an overall asymmetry of the complex structure stemming from the deprotonation of one of the oxime termini. The structure of the Ni(II) ion coordination environment is similar for both complexes, with shorter Ni−N_ad_ bond distances for the RNC, which may be a reflection of a more polarisable electron density in the conjugated system of the propene spacer and easier donation to the Ni acceptor orbitals. As expected, the N_ad_−C bonds are much shorter for the RNC than for the YNC and, in fact, are representative of the C=N bond. There is only a slight difference between the C−C bond lengths of the propene spacer, which confirms a delocalised π-conjugated structure of the bridge. The C(=O)−N_ad_ bonds are noticeably shorter, while C=O bonds are noticeably longer for the YNC core, which is an indication of partial donation of the electron density from the carbonyl bond to the amide nitrogen and further to the Ni(II) centre. In the RNC, this effect is diminished due to the stronger influence of the conjugated π-system of the spacer. The N2−Ni−N3 bond angle, where the spacer is attached to two nitrogen atoms, is smaller by 2° for the RNC, and is a reflection of the higher stiffness of the conjugated propene bridge in comparison to the propane bridge. The length of the hydrogen bond between the two oxime termini that locks the pseudo-macrocyclic structure of the complex, O4⋯H1, is longer by 0.088 Å for the RNC than for the YNC; the latter is also a reflection of a more rigid spacer that joins two chelating oximato–amide and oxime–ketoimine moieties.

Comparison of the computed and experimental XRD structures, shown in Table 6 and Table 7, affords the following observations. There is more than one set of geometric parameters for each complex core in the solid state, due to different polymorphic structures or different sites occupied by the core in the crystal lattice, as is the case for (**8**). Understandably, the molecular structure is affected by the crystal lattice interactions. In general, there is close agreement between the computed-in-MeOH medium and solid-state XRD structures, as shown in Supplementary Materials Tables S8 and S9, with the latter being somewhat more symmetrical, perhaps, due to the averaging effect of the lattice forces. All structural effects noted above for the computed structures are also observed for the solid-state structures.

The UV–Vis spectra of the complexes were calculated by means of time-dependent DFT (TD-DFT), where the end result was an excitation energy and a set of wavefunction coefficients describing the contribution of each particle-hole pair to the given excited state. In layman’s terms, the method considers a multitude of one-electron adiabatic transitions from a certain occupied molecular orbital to a certain empty molecular orbital, with the transition wavefunction being a resonance of a number of such steps. Conventional molecular orbitals, CMOs, have rather complex shapes, and are difficult to interpret in the bonding terms favoured by chemists. Thus, it is preferential to use natural bond orbitals, NBOs, a unique set of orthonormal single-electron localised functions closest to Lewis types of one-centre (“lone pairs”) and two-centre (“bonds”) orbitals, which allow for the conventional chemist’s interpretation of bonding behaviour in molecules. In some instances, a certain particle-hole coefficient significantly exceeds all others; in such cases, the nature of the transition states is clearly defined. More often, a few orbital coefficients are comparable in size; in such cases the transition states are less obvious, as they represent a resonance of a few states. However, even in such cases, it is beneficial to employ NBOs, and in many instances, the transition states could be assigned reasonable physical meaning.

As can be seen from our results, quantum chemical modelling is indeed capable of predicting the colour (absorption spectrum) of Ni complexes close to those observed experimentally; moreover, it allows elucidation of the nature of the electronic transitions responsible for such colours. In particular, the absorption spectrum of YNC is dominated by two transitions at 302 nm and 356 nm, which remove most of the ultra-violet, violet, blue, and some of the green part of the spectrum, resulting in the yellow hue reflected by the solid surface, or light transmitted through the solution. The first transition, in terms of NBOs, primarily represents electron excitation from the amide oxygen $p_{z}^{2}$ lone pair orbital into the oxime C=N antibonding $\pi^{*}$ orbital. The second transition primarily represents an electron transfer from the amide nitrogen $p_{z}^{2}$ lone pair orbital to the same oxime C=N antibonding $\pi^{*}$ orbital. Thus, both transitions account for the electron transfer within the ligand scaffold from the amide to the oxime functional group.

In contrast, the absorption spectrum of the RNC is more complex, and requires considering at least four spectral lines of noticeable amplitude, with wavelengths of 324 nm, 392 nm, 488 nm, and 493 nm, as shown in Figure 11, and Supplementary Materials Table S6. These transitions effectively remove most of the visible part of incident light, resulting in the red hue reflected by the surface of this complex, or light transmitted through its solution. A fundamental difference of this case from the previous one is the involvement of Ni(II) lone pair *d*-orbitals in all of the above transitions. Splitting of the 3*d*^8^ Ni energy levels in the square-planar ligand field results in two degenerate lowest energy orbitals, $d_{xz}^{2}\;and\;d_{yz}^{2},$ and three more orbitals of progressively higher energy, $d_{z^{2}}^{2}$, $d_{xy}^{2}$, and $d_{x^{2}-y^{2}}^{0}$. According to our calculations, considerable contribution to all four spectroscopically active excited states comes from the electron transfers, which originate from the three bottom nickel 3*d* orbitals and proceed into the antibonding $\pi^{*}$ orbitals localised on the oxime, amide, and ketoimine groups. In other words, major absorption lines in the spectrum of this complex are caused, at least in part, by the typical metal-to-ligand charge transfer (MLCT). A feature of the computed electronic structure of the RNC to be mentioned is the $p_{z}^{2}$ orbital on the central carbon atom, C9, of the spacer, which contributes noticeably to all three spectral lines at longer wavelengths. In particular, the electron transfers from this orbital into antibonding $\pi^{*}\;$ orbitals located on the oxime N1–C2, N4–C7, and imine N2−C4 centres, which feature prominently at 392 nm, 488 nm, and 493 nm, respectively.

An opinion expressed in the literature was that the Ni–Ni interactions give rise to an absorption band in the visible region of the spectrum, and thus may account for the red colour of various Ni(II) *vic*-dioxime complexes [20]. The experimental wavelength of maximum absorbance for these complexes was reported in the range of 465 nm to 554 nm. It was also stipulated that reduction in the Ni–Ni distance, e.g., by ligand design or increased pressure, causes red shift in the spectrum [20,25]. By analogy, the same argument could be extended to our RNC. Although we do not reject the idea that Ni–Ni interactions in the stacked complexes may contribute to the electronic spectrum, we are doubtful that such contributions are of primary importance. Our reasoning is based on the following. Firstly, to the naked eye, the red colour of both Ni(*dmg*)_2_ and the RNC does not change significantly upon dissolution. Obviously, one cannot expect preservation of the stacked metal columns of any length in solvent media. Secondly, the reported $[{{Ni(HAO)}_{2}]}^{0}$ complex of the doubly deprotonated *α*-hydroxylamine oxime ligand is deep purple in colour (λ_max_ = 541 nm), while the Ni–Ni distance in the stack is 6.400 Å [29]; consequently, such interactions cannot contribute to the visible spectrum. Thirdly, we carried out our quantum chemical modelling on a single complex molecule, without accounting for any Ni–Ni interactions (modelling a cluster of stacked molecules is much more challenging), and obtained a simulated spectrum that is in reasonable agreement with the experimental one. Thus, in contrast to the mentioned opinion, we think that the red colour of such complexes is caused by intramolecular transitions, rather than by transitions into Ni(II) virtual orbitals that are affected by the metal–metal bond length. In particular, the analysis of spectral transitions in the visible region of the spectrum presented above indicates that all of them are of the MLCT nature rather than transitions into Ni 4*p*_z_ vacant orbitals that are predominantly stabilised by the shortening of the Ni–Ni bond in the stacked structure.

## 4. Materials and Methods

### 4.1. Materials

Organic solvents, reagents, and other materials were purchased from commercial suppliers and were of analytical or reagent grade. They were used without further purification. New complexes were synthesised as reported in Supplementary Materials Synthesis S1–S8.

### 4.2. Instrumental

**IR:** FTIR spectra were recorded in KBr disks on a Perkin Elmer Spectrum 100 spectrometer in the range of 450 to 4000 cm^−1^ with a resolution of 1 cm^−1^, or with unmodified sample material on a Bruker Alpha-II Platinum ATR spectrometer in the range of 400 to 4000 cm^−1^ with a resolution of 4 cm^−1^.

**CHN:** Elemental analyses were performed in the Laboratorio di Microanalisi, University of Florence (Italy).

**NMR:** ^1^H, ^13^C, ^15^N, and ^31^P NMR spectra were recorded on a Bruker Avance-III 400 or Bruker Avance-III 500 spectrometer at frequencies of 400/500 MHz (^1^H) and 100/125 MHz (^13^C), using either a 5 mm BBOZ-[^31^P-^109^Ag]-{^1^H} probe or a 5 mm TBIZ-[^1^H]-{^31^P}-{^31^P-^109^Ag} probe. All proton and carbon chemical shifts are quoted relative to the relevant solvent signal (e.g., for DMSO-*d*_6_, ^1^H: 2.50 ppm, ^13^C: 39.50 ppm). Coupling constants are reported in Hertz (Hz). All of the experiments were conducted at 30 °C.

**MS–ToF:** High-resolution mass spectra were recorded on a Waters UPLC Acquity—Micromass LCT Premier ToF/MS spectrometer. The samples were dissolved in DMSO to a concentration of approximately 2 mg L^−1^. For low-resolution measurements, the instrument was internally calibrated with either reserpine (positive ionisation mode) or raffinose (negative ionisation mode). High-resolution measurements were performed using DMSO as the lock mass standard. Pure samples were injected directly into the MS port, i.e., bypassing the UPLC system.

**UV–Vis:** UV–Vis spectra of methanolic solutions were recorded against pure solvent in a 10 mm light path transmission-matched pair of precision Hellma Analytics Quartz SUPRACIL cells on a Shimadzu-1280 spectrometer. The spectra were recorded in the range of 190 nm to 1100 nm, with a slit width of 5 nm, a “medium” scan rate of 180 nm min^−1^ in two consecutive runs, with the results averaged. The concentration of complex solutions was chosen so that the value of absorption at λ_max_ was close to 1.2.

**XRD:** Reflection data were acquired on single-crystal diffractometers XcaliburPX Ultra or Xcalibur3 (Oxford Diffraction). The integrated intensities were corrected for Lorentzian and polarisation effects, and an empirical absorption correction was applied, SCALE3 ABSPACK [31]. Crystal structures were solved via direct methods with SIR97 [32], and refinements were performed by means of full-matrix least-squares using SHELXL (version 2019/2) [33]. Non-hydrogen atoms were refined anisotropically, while riding models were used for all the hydrogen atoms, with the exception of oxygen-bound hydrogens. Additional details of refinement for each single structure are shown in Supplementary Materials SR S1–S8. Mercury 2023.1.0 (Build 376230) [34,35], enCIFer 2023.1.0 (Build 376230) [36], Olex2-1.5 [37], and ORTEP-3 (version 1.076, 2020) [38] were used for processing, visualisation, and presentation of structural data. New structures were deposited into the CCDC [39], with reference code numbers **2285820–2285826**.

**QM:** Quantum chemical modelling was performed using the Gaussian 09W suite of software [40] and the GaussView 5.0 interface, on an 8-core CPU Windows-64 platform. The structures and spectra were computed using the density functional theory (DFT) method, with the restricted B3LYP hybrid functional and split-valence 6-311 basis set augmented with diffuse and polarised functions. Molecular systems in solution were treated using a self-consistent reaction field method in the form of a polarised continuum model, IEFPCM [41]. Standard solvent parameters were used in calculations, in particular, the value $\varepsilon_{r}=32.613$ for “the relative dielectric constant” of methanol. Natural bond orbitals, NBOs, were computed within Gaussian 09W according to F. Weinhold and coworkers [42]. The calculations consisted of a sequence of steps. In all cases, the B3LYP DFT functional was used for calculation of a singlet electronic state. First, a sound conformer choice based on chemical intuition, previous experience, and solid-state structure, was optimised with the 6-31+G (d, p) basis set in vacuo, step *G1*. Next, the obtained structure was further optimised in vacuo with a larger basis set—6-311++G (3d, 2p), step *G2*. The G2 wavefunction was tested for stability, step *G2-Stab*, after which the structure from step G2 was optimised with the same basis set in a solvent (methanol) medium, step *S2*. Methanol was chosen as the solvent because both metal complexes of interest are very soluble in it. The S2 wavefunction was also tested for stability, step *S2-Stab*. Finally, the UV–Vis spectrum was computed in a single-point energy calculation using the TD-SCF version of DFT [43] for 12 excited states, still in MeOH medium, with a full set of natural bond orbitals (NBOs), step *S2-TD-NBO*. The keywords and options used for each Gaussian step are shown below:

**G1:** # opt = (calcfc, tight) b3lyp/6-31+g (d, p)

**G2:** # opt = (calcfc, tight) b3lyp/6-311++g (3d, 2p)

**G2-Stab:** # stable b3lyp/6-311++g (3d, 2p)

**S2:** # opt = (calcfc, tight) b3lyp/6-311++g (3d, 2p) scrf = (iefpcm, solvent = methanol)

**S2-Stab:** # stable b3lyp/6-311++g (3d, 2p) scrf = (iefpcm, solvent =m ethanol)

**S2-TD-NBO:** # td = (nstates = 12) b3lyp/6-311++g (3d, 2p) scrf = (iefpcm, solvent = methanol) pop = (nbo, savenbo).

## 5. Conclusions

Seven new Cu(II) and Ni(II) coordination compounds with the *bis*-chelate oxime–amide/ketoimine ligands were synthesised and characterised. The full range of FTIR, NMR, and MS–ToF spectra were recorded for one more Ni(II) complex, ${\left[{PPh}_{4}\right]}^{+}\cdot {\left[Ni\left(II\right)L2H_{-3}\right]}^{-}\cdot H_{2}O,$ for which the synthesis and crystal structure have previously been reported, but no other relevant characterisation data were provided.

Molecular and crystal structures for all new coordination compounds determined by single crystal X-ray diffraction were presented, together with a comparative analysis of structural features.

The highlight of this research was the discovery of a novel type of neutral Ni(II) complex with the *bis*-chelate oxime–amide/ketoimine ligand. These planar complexes assemble in the solid state on top of each other in a structure that resembles a stack of poker chips, and form infinite, exclusively parallel metal ion columns in ligand insulation with all Ni ions directly bound to each other in essentially one-dimensional rods—a rare combination of structural features for Ni(II) coordination compounds. Two such polymorphic structures were reported with different turn angles of their neighbouring complexes in the stack, and different sets of Ni−Ni distances.

To interpret the difference in colour between the structurally similar yellow and red Ni(II) complexes, their experimental and quantum chemically computed UV–Vis absorption spectra, as well as interpretation of the nature of their electronic transitions and the shapes of the natural bond orbitals involved in them were presented. According to our results, the red colour of the Ni(II) complexes is not associated with transitions into vacant nickel 4*p*_z_ orbitals that are primarily stabilised by the shortening of Ni–Ni distances in the solid-state stacked structure; instead, it is caused by intramolecular transitions that are mainly MLCT in nature.

## Figures and Tables

**Table 1:** Cu(II) complexes reported in this paper.

Ligand	Coordination Mode	Complex
L1	2(N_ox_O_ad_)	$[\{Cu(II)L1H_{-1}{\}}^{+}(OH_{2}){]}_{2}^{2+}\cdot 2NO_{3}^{-}\cdot {2H}_{2}O$ = (**1**) sp-tpp ^1^
L3	2(N_ox_O_ad_)	$[\{Cu\left(II\right)L3H_{-1}{\}}^{+}(OH_{2}){]}_{2}^{2+}\cdot 2BF_{4}^{-}\cdot {2H}_{2}O$ = (**2**) sp-tpp
L3	2(N_ox_O_ad_)	$[\{Cu\left(II\right)L3H_{-1}{\}}^{+}{{Cl}^{-}]}_{2}^{0}\cdot {4H}_{2}O$ = (**3**) sp-tpp
L3	2(N_ox_O_ad_)	$[\{Cu\left(II\right)L3H_{-1}{\}}^{+}{Cl}^{-}{]}_{2}^{0}\cdot {L3\cdot 2H}_{2}O$ = (**4**) sp-tpp
L2	2(N_ox_N_ad_)	${\left[{\left\{Li{\left({OH}_{2}\right)}_{3}\right\}}^{+}{\cdot \{Cu\left(II\right)L2H_{-3}\}}^{-}\right]}^{0}\cdot H_{2}O$ = (**5**) ^2^ sppm ^3^

^1^ Sp-tpp stands for *square-pyramidal two parallel platform* structure. ^2^ In this structure, a thrice-hydrated ${\left\{Li{\left({OH}_{2}\right)}_{3}\right\}}^{+}$ ion is coordinated to the amide oxygen of the anionic sppm complex, forming a neutral unit. ^3^ Sppm stands for *square-planar pseudo-macrocyclic* structure.

**Table 2:** Ni(II) complexes reported in this paper.

Ligand	Coordination Mode	Complex
L2	2(N_ox_N_ad_)	${\left[{PPh}_{4}\right]}^{+}\cdot {\left[Ni\left(II\right)L2H_{-3}\right]}^{-}{\cdot H}_{2}O$ = (**6**) ^1^ sppm
L2′	(N_ox_N_ad_)(N_ox_N_ki_)	${\left({\left[Ni\left(II\right)L2^{\prime }H_{-2}\right]}^{0}\right)}_{n}\left(\alpha \right)$ = (**7**) ^2^ dohp ^3^
L2′	(N_ox_N_ad_)(N_ox_N_ki_)	${\left({{\{\left[Ni\left(II\right)L2^{\prime }H_{-2}\right]}^{0}\}}_{2}\right)}_{n}\left(\beta \right)$ = (**8**) ^2^ dohdp ^4^

^1^ The crystal structure of this complex turned out to be the same as reported in [14]. ^2^ (**7**) and (**8**) are polymorphs, i.e., they have the same molecular structure but different crystal structures. ^3^ Dohp stands for *distorted octahedral polymer*, where neutral complexes form a “poker chips” stack with equidistant separation of all Ni(II) ions. ^4^ Dohdp stands for *distorted octahedral dimer polymer*, where two neutral complexes form a dimeric pair of one Ni–Ni distance, and such pairs stack in “poker chips” columns with a different Ni–Ni distance between them.

**Table 3:** Crystal data and structure refinement parameters for complexes (**1**)–(**5**), (**7**)–(**8**).

Complex	(1)	(2)	(3)	(4)	(5)	(7)	(8)
Empirical formula	C_16_H_32_N_10_O_17_Cu_2_	C_20_H_42_N_8_O_12_B_2_F_8_Cu_2_	C_20_H_42_N_8_O_12_Cl_2_Cu_2_	C_30_H_56_N_12_O_14_Cl_2_Cu_2_	C_9_H_21_N_4_O_8_CuLi	C_9_H_10_N_4_O_4_Ni	C_9_H_10_N_4_O_4_Ni
*M*	763.57	887.30	784.59	1006.84	383.77	296.89	296.89
*T*/K	293 (2)	293(2)	293(2)	293(2)	293 (2)	293(2)	293(2)
Crystal system	monoclinic	triclinic	triclinic	triclinic	triclinic	monoclinic	triclinic
Space group	*I* 2/*a*	*P* $\bar{1}$	*P* $\bar{1}$	*P* $\bar{1}$	*P* $\bar{1}$	*C c*	*P* $\bar{1}$
*a*/Å	8.027(1)	8.664(5)	8.141(1)	8.8321(1)	7.450(2)	9.5938(13)	6.5672(6)
*b*/Å	23.663(3)	9.516(5)	8.887(2)	10.8660(1)	8.862(1)	19.587(2)	10.5171(10)
*c*/Å	15.194(5)	11.745(5)	11.414(2)	11.8382(2)	11.922(2)	6.5058(8)	16.1337(14)
*α*/°	90	102.016(5)	95.47(1)	105.766(1)	103.19(1)	90	85.084(7)
*β*/°	96.135(14)	91.879(5)	94.41(1)	96.551(2)	97.31(1)	117.346(9)	82.041(7)
*γ*/°	90	108.828(5)	102.823(5)	100.627(2)	90.39(1)	90	80.262(7)
*V*/Å^−3^	2869.5(10)	891.2(8)	797.4(2)	1058.30(3)	759.6(3)	1085.9(2)	1085.47(17)
*Z*	4	1	1	1	2	4	4
*d*_c_/g cm^−3^	1.768	1.653	1.634	1.580	1.678	1.816	1.817
*μ*/mm^−1^	1.577	1.300	1.570	1.208	2.481	2.757	1.801
*F*(0 0 0)	1568	454	406	524	398	608	608
Crystal size /mm	0.1 × 0.05 × 0.05	0.2 × 0.15 × 0.15	0.15 × 0.15 × 0.1	0.23 × 0.18 × 0.1	0.25 × 0.1 × 0.1	0.22 × 0.15 × 0.1	0.17 × 0.13 × 0.1
Colour	dark green	dark green	dark green	dark green	red	raspberry red	wine red
λ/Å	0.71069	0.71069	0.71069	0.71069	1.5418	1.5418	0.71069
*θ* range/°	4.137 to 22.541	4.130 to 28.431	3.884 to 28.370	1.999 to 25.871	5.130 to 58.947	4.515 to 72.105	3.815 to 27.503
Index range	−6 ≤ h ≤ 8,	−11 ≤ h ≤ 11,	−10 ≤ h ≤ 10,	−10 ≤ h ≤ 10,	−8 ≤ h ≤ 8,	−11 ≤ h ≤ 9,	−8 ≤ h ≤ 8,
	−21 ≤ k ≤ 25,	−12 ≤ k ≤ 12,	−10 ≤ k ≤ 11,	−12 ≤ k ≤ 13,	−9 ≤ k ≤ 8,	−23 ≤ k ≤ 22,	−13 ≤ k ≤ 12,
	−16 ≤ l ≤ 16	−14 ≤ l ≤ 15	−15 ≤ l ≤ 14	−14 ≤ l ≤ 14	−13 ≤ l ≤ 13	−6 ≤ l ≤ 7	−17 ≤ l ≤ 20
Reflections collected	5734	9350	8239	19106	5677	2091	9259
Independent Reflections/Rint, Rsigma	1886/ 0.0662, 0.117	3807/ 0.0281, 0.0421	3408/ 0.0371, 0.075	3972/ 0.0263, 0.0183	2109/ 0.0350, 0.0318	1291/ 0.0443, 0.0376	4162/ 0.0371, 0.0897
Data/ restraints/ parameters	1886/0/211	3807/0/292	3408/0/207	3972/1/283	2109/0/218	1291/149/166	4162/0/343
Goodness of fit on *F*^2^	0.817	1.052	0.927	0.978	0.973	1.086	0.872
*R* indices							
[*I* > 2σ(*I*)]							
*R*_1_	0.0501	0.0384	0.0334	0.0370	0.0424	0.0419	0.0350
*wR*_2_	0.1020	0.0935	0.0800	0.1006	0.1147	0.1121	0.0754
*R* indices							
(all data)							
*R*_1_	0.0927	0.0530	0.0548	0.0399	0.0499	0.0467	0.0696
*wR*_2_	0.1150	0.1016	0.0767	0.1043	0.1203	0.1197	0.0804
Max/min electron density/e Å^−3^	0.73/−0.44	0.98/−0.37	0.66/−0.48	0.59/−0.93	0.65/−0.76	0.39/−0.81	0.67/−0.39

**Table 4:** Selected values of bond lengths (Å) and angles (°) for cationic Cu(II) complexes with axial water or chloride coordination in crystal structures (**1**)–(**4**). Formally, complexes (**3**) and (**4**) are neutral; however, for the reasons of structural similarity with (**1**) and (**2**), we consider them in the class of cationic complexes.

(1)		(2)		(3)		(4)	
Cu N1	1.960(5)	Cu N1	1.957(2)	Cu N1	1.960(2)	Cu N1	1.969(3)
Cu N4	1.946(5)	Cu N4	1.939(2)	Cu N4	1.958(2)	Cu N4	1.964(2)
Cu O2	1.962(4)	Cu O2	1.984(2)	Cu O2	1.974(2)	Cu O2	1.988(2)
Cu O3	1.978(4)	Cu O3	1.963(2)	Cu O3	1.972(2)	Cu O3	1.983(2)
Cu OW1	2.194(5)	Cu OW1	2.175(3)	Cu Cl	2.4624(8)	Cu Cl	2.5678(8)
C3 O2	1.267(7)	C3 O2	1.258(3)	C3 O2	1.266(3)	C3 O2	1.264(3)
C6 O3	1.261(8)	C6 O3	1.265(3)	C6 O3	1.267(3)	C6 O3	1.266(3)
N1 C2	1.29(1)	N1 C2	1.283(3)	N1 C2	1.272(3)	N1 C2	1.285(3)
N4 C7	1.283(8)	N4 C7	1.290(3)	N4 C7	1.291(3)	N4 C7	1.292(3)
O1 N1	1.331(8)	O1 N1	1.354(3)	O1 N1	1.358(3)	O1 N1	1.352(3)
O4 N4	1.371(6)	O4 N4	1.332(3)	O4 N4	1.331(3)	O4 N4	1.331(3)
C3 N2	1.330(9)	C3 N2	1.318(4)	C3 N2	1.303(3)	C3 N2	1.320(3)
C6 N3	1.332(8)	C6 N3	1.317(3)	C6 N3	1.303(3)	C6 N3	1.316(4)
O1 O4	2.500	O1 O4	2.495	O1 O4	2.500	O1 O4	2.470
O1 H1	1.226	O1 H1	1.040	O1 H1	1.032	O1 H1	0.885
O4 H1	1.282	O4 H1	1.459	O4 H1	1.476	O4 H1	1.588
BP ^1^—Cu	0.2458(8)	BP—Cu	0.2010(4)	BP—Cu	0.3200(4)	BP—Cu	0.2247(4)
N1 Cu O2	81.8(2)	N1 Cu O2	80.50(8)	N1 Cu O2	80.38(8)	N1 Cu O2	81. 03(10)
O2 Cu O3	99.1(2)	O2 Cu O3	99.07(8)	O2 Cu O3	96.99(7)	O2 Cu O3	99.98(8)
O3 Cu N4	80.6(2)	O3 Cu N4	81.96(8)	O3 Cu N4	81.27(8)	O3 Cu N4	81.64(9)
N4 Cu N1	94.9(2)	N4 Cu N1	96.06(9)	N4 Cu N1	95.25(9)	N4 Cu N1	94.4(1)
N1 Cu O3	165.0(2)	N1 Cu O3	169.26(9)	N1 Cu O3	162.30(8)	N1 Cu O3	166.73(9)
O2 Cu N4	165.9(2)	O2 Cu N4	166.98(8)	O2 Cu N4	160.17(8)	O2 Cu N4	166.54(9)
N1 Cu OW1	101.6(2)	N1 Cu OW1	94.40(10)	N1 Cu Cl	98.87(7)	N1 Cu Cl	96.65(7)
O2 Cu OW1	101.1(2)	O2 Cu OW1	93.85(9)	O2 Cu Cl	99.65(5)	O2 Cu Cl	95.23(6)
O3 Cu OW1	93.0(2)	O3 Cu OW1	96.33(9)	O3 Cu Cl	98.83(5)	O3 Cu Cl	96.43(6)
N4 Cu OW1	93.1(2)	N4 Cu OW1	98.96(9)	N4 Cu Cl	100.13(7)	N4 Cu Cl	97.88(7)
BP Cu-OW1	84.5(1)	BP Cu-OW1	87.51(8)	BP Cu-Cl	89.86(4)	BP Cu-Cl	88.69(4)

^1^ BP stands for the *basal plane*—an average plane drawn through four donor atoms N1-O2-O3-N4 of two chelating ligands.

**Table 5:** Selected values of bond lengths (Å) and angles (°) for an anionic Cu(II) complex in crystal structure (**5**).

(5)							
Cu N1	1.963(2)	N1 C2	1.273(4)	O1 O4	2.580	N1 Cu N2	81.6(1)
Cu N2	1.930(3)	N4 C7	1.281(4)	O1 H1	0.839	N2 Cu N3	99.2(1)
Cu N3	1.920(2)	O1 N1	1.384(4)	O4 H1	1.751	N3 Cu N4	82.5(1)
Cu N4	1.953(3)	O4 N4	1.374(3)	O2 Li	1.953(6)	N4 Cu N1	96.49(11)
C3 O2	1.251(4)	C3 N2	1.325(4)	BP—Cu	0.0658(5)	N1 Cu N3	174.5(1)
C6 O3	1.271(3)	C6 N3	1.296(4)			N2 Cu N4	177.0(1)

**Table 6:** Selected values of bond lengths (Å) and angles (°) for the neutral RNC in crystal structures (**7**) and (**8**).

(7)		(8)			
Ni N1	1.877(8)	Ni1 N1	1.858(3)	Ni2 N5	1.867(2)
Ni N2	1.861(7)	Ni1 N2	1.841(3)	Ni2 N6	1.848(2)
Ni N3	1.851(8)	Ni1 N3	1.842(3)	Ni2 N7	1.837(2)
Ni N4	1.873(8)	Ni1 N4	1.862(3)	Ni2 N8	1.875(3)
C3 O2	1.228(11)	C3 O2	1.193(4)	C12 O6	1.200(4)
C6 O3	1.210(13)	C6 O3	1.207(4)	C15 O7	1.205(3)
N1 C2	1.282(11)	N1 C2	1.294(4)	N5 C11	1.293(4)
N4 C7	1.274(13)	N4 C7	1.293(4)	N8 C16	1.297(4)
O1 N1	1.339(10)	O1 N1	1.348(3)	O5 N5	1.343(3)
O4 N4	1.342(10)	O4 N4	1.350(3)	O8 N8	1.335(3)
C3 N2	1.441(11)	C3 N2	1.421(4)	C12 N6	1.413(4)
C6 N3	1.412(11)	C6 N3	1.418(4)	C15 N7	1.427(4)
N2 C4	1.315(14)	N2 C4	1.339(4)	N6 C13	1.331(4)
N3 C5	1.325(13)	N3 C5	1.331(4)	N7 C14	1.326(4)
C4 C9	1.369(14)	C4 C9	1.383(4)	C13 C18	1.391(4)
C5 C9	1.406(12)	C5 C9	1.377(5)	C14 C18	1.380(5)
O1 O4	2.439	O1 O4	2.449	O5 O8	2.446
O1 H1	1.024	O1 H1	0.992	O5 H2	1.125
O4 H1	1.415	O4 H1	1.472	O8 H2	1.340
BP—Ni	0.010(2)	BP—Ni1	0.0047(4)	BP—Ni2	0.0017(4)
N1 Ni N2	84.0(3)	N1 Ni1 N2	83.51(11)	N5 Ni2 N6	83.15(11)
N2 Ni N3	94.4(4)	N2 Ni1 N3	95.22(11)	N6 Ni2 N7	94.83(11)
N3 Ni N4	83.7(3)	N3 Ni1 N4	83.40(11)	N7 Ni2 N8	84.20(11)
N4 Ni N1	97.8(3)	N4 Ni1 N1	97.88(12)	N8 Ni2 N5	97.82(12)
N1 Ni N3	178.2(4)	N1 Ni1 N3	178.54(11)	N5 Ni2 N7	177.95(12)
N2 Ni N4	178.0(5)	N2 Ni1 N4	178.61(11)	N6 Ni2 N8	179.02(11)

**Table 7:** Selected bond lengths (Å) and valence angles (°) of the computed complex cores.

YNC				RNC			
Ni−N1	1.903	C7−C6	1.502	Ni−N1	1.905	C7−C6	1.473
Ni−N4	1.900	N2−C4	1.453	Ni−N4	1.895	N2−C4	1.322
Ni−N2	1.898	N3−C5	1.455	Ni−N2	1.880	N3−C5	1.321
Ni−N3	1.883	C4−C9	1.527	Ni−N3	1.864	C4−C9	1.387
O1−N1	1.354	C5−C9	1.528	O1−N1	1.340	C5−C9	1.398
O4−N4	1.324	O1−O4	2.461	O4−N4	1.257	O1−O4	2.486
C3−O2	1.246	O1−H1	1.067	C3−O2	1.216	O1−H1	1.049
C6−O3	1.248	O4⋯H1	1.402	C6−O3	1.217	O4⋯H1	1.449
N1−C2	1.283	N1−Ni−N2	82.60	N1−C2	1.287	N1−Ni−N2	83.00
N4−C7	1.292	N4−Ni−N3	83.57	N4−C7	1.304	N4−Ni−N3	83.93
C3−N2	1.334	N1−Ni−N4	96.59	C3−N2	1.399	N1−Ni−N4	97.85
C6−N3	1.339	N2−Ni−N3	97.23	C6−N3	1.417	N2−Ni−N3	95.22
C1−C2	1.488	N1−Ni−N3	179.28	C1−C2	1.486	N1−Ni−N3	178.33
C8−C7	1.489	N4−Ni−N2	179.02	C8−C7	1.488	N4−Ni−N2	179.15
C2−C3	1.513	O1−H1⋯O4	170.04	C2−C3	1.498	O1−H1⋯O4	168.35

## Data Availability

The data for all new structures have been deposited into the CSD [39] with reference code numbers 2285820–2285826.

## Supplementary Materials

The following supporting information can be downloaded at: https://www.mdpi.com/article/10.3390/molecules29020522/s1, Table S1. Ni(II) complexes reported in literature; Table S2. Cu(II) complexes reported in literature; Table S3. NMR coupling constants derived for the tetraphenylphosphonium cation in DMSO-*d*_6_ solvent at 30 °C; Table S4. Selected values of the excited state parameters computed for the YNC core; Table S5. Computed NBOs and their parameters for the YNC core; Table S6. Selected values of the excited-state parameters computed for RNC; Table S7. Computed NBOs and their parameters for RNC; Table S8. Comparison of the selected bond lengths (Å) and valence angles (°) for the experimental (XRD, (**6**) [14]) and computed structure of YNC core; Table S9. Comparison of the selected bond lengths (Å) and valence angles (°) for the experimental (XRD, (**7**)) and computed structure of RNC. Scheme S1. A segment of tetraphenylphosphonium cation with the labelling scheme used for the assignment of NMR coupling constants in this compound. Figure S1. FTIR spectrum of (**1**); Figure S2. FTIR spectrum of (**2**); Figure S3. FTIR spectrum of (**5**); Figure S4. MS–ToF spectrum of (**5**); Figure S5. FTIR spectra of (**6**). Top: Spectrum 100. Bottom: Alpha II platinum; Figure S6. ^1^H NMR spectrum of (**6**); Figure S7. A fragment of ^1^H NMR spectrum for YNC representing resonances attributable to the tetraphenylphosphonium (TPP) cation; Figure S8. GCOSY NMR spectrum of (**6**); Figure S9. ^13^C-{^1^H} NMR spectrum of (**6**); Figure S10. ^13^C-{^1^H} NMR spectrum of the TPP cation in (**6**); Figure S11. DEPT-135 NMR spectrum of (**6**); Figure S12. GHSQC NMR spectrum of (**6**); Figure S13. GHMBC NMR spectrum of (**6**); Figure S14. ^15^N projection of GHMBC NMR spectrum of (**6**); Figure S15. GHMBC-^15^N NMR spectrum of (**6**); Figure S16. ^31^P NMR spectrum of (**6**); Figure S17. Experimental (top) and simulated (bottom) ^1^H spectra of the tetraphenylphosphine (TPP) cation; Figure S18. MS–ToF spectrum of (**6**); Figure S19. FTIR spectra of (**7**); Figure S20. FTIR spectra of (**8**); Figure S21. ^1^H NMR spectrum of (**7**); Figure S22. GCOSY NMR spectrum of (**7**); Figure S23. ^13^C-{^1^H} NMR spectrum of (**7**); Figure S24. GHSQC NMR spectrum of (**7**); Figure S25. GHMBC NMR spectrum of (**7**); Figure S26. ^15^N projection of GHMBC NMR spectrum of (**7**); Figure S27. GHMBC-^15^N NMR spectrum of (**7**); Figure S28. MS–ToF spectrum of (**7**); Figure S29. Computed NBOs involved in the electron transitions that account for the UV–Vis spectrum of YNC (left) and RNC (right); Figure S30. Computed energy diagram for YNC (left) and RNC (right). Bonding and antibonding states are separated by the dashed line. Synthesis: Synthesis S1. (**1**) = $[\{Cu(II)L1H_{-1}{\}}^{+}(OH_{2}){]}_{2}^{2+}\cdot 2NO_{3}^{-}\cdot H_{2}O$; Synthesis S2. (**2**) = $[\{Cu\left(II\right)L3H_{-1}{\}}^{+}(OH_{2}){]}_{2}^{2+}\cdot 2BF_{4}^{-}\cdot {2H}_{2}O$; Synthesis S3. (**3**) = $[\{Cu\left(II\right)L3H_{-1}{\}}^{+}{Cl}^{-}{]}_{2}^{0}\cdot {4H}_{2}O$; Synthesis S4. (**4**) = $[\{Cu\left(II\right)L3H_{-1}{\}}^{+}{Cl}^{-}{]}_{2}^{0}\cdot {L3\cdot 2H}_{2}O$; Synthesis S5. (**5**) = ${\left[{\left\{Li{\left({OH}_{2}\right)}_{3}\right\}}^{+}{\left\{Cu\left(II\right)L2H_{-3}\right\}}^{-}\right]}^{0}\cdot H_{2}O$; Synthesis S6. (**6**) = ${{\left[{PPh}_{4}\right]}^{+}\cdot \left[Ni\left(II\right)L2H_{-3}\right]}^{-}\cdot H_{2}O$; Synthesis S7 and S8. (**7**) and (**8**) = ${\left[Ni\left(II\right)L2^{\prime }H_{-2}\right]}^{0}$. Structure Refinement: SR S1. (**1**) = $[\{Cu(II)L1H_{-1}{\}}^{+}(OH_{2}){]}_{2}^{2+}\cdot 2NO_{3}^{-}\cdot H_{2}O$; SR S2. (**2**) = $[\{Cu\left(II\right)L3H_{-1}{\}}^{+}(OH_{2}){]}_{2}^{2+}\cdot 2BF_{4}^{-}\cdot {2H}_{2}O$; SR S3. (**3**) = $[\{Cu\left(II\right)L3H_{-1}{\}}^{+}{Cl}^{-}{]}_{2}^{0}\cdot {4H}_{2}O$; SR S4. (**4**) = $[\{Cu\left(II\right)L3H_{-1}{\}}^{+}{Cl}^{-}{]}_{2}^{0}\cdot {L3\cdot 2H}_{2}O$; SR S5. (**5**) = ${\left[{\left\{Li{\left({OH}_{2}\right)}_{3}\right\}}^{+}{\left\{Cu\left(II\right)L2H_{-3}\right\}}^{-}\right]}^{0}\cdot H_{2}O$; SR S7. (**7**) = ${\left[Ni\left(II\right)L2^{\prime }H_{-2}\right]}^{0}$; SR S8. (**8**) = ${\left[Ni\left(II\right)L2^{\prime }H_{-2}\right]}^{0}$.
