# Supplementary material for: Old Acquaintances and Novel Complex Structures for the Ni(II) and Cu(II) Complexes of bis-Chelate Oxime–Amide Ligands
Source: Molecules. 2024 Jan 20;29(2):522. doi: 10.3390/molecules29020522 (PMC10819512; doi:10.3390/molecules29020522)
Supplement: Supplementary file 1 [file molecules-29-00522-s001.zip › molecules-2807321-supplementary.pdf]

molecules

Article

Old Acquaintances and Novel Complex Structures for the Ni(II) and Cu(II) Complexes of *bis*-Chelate Oxime-Amide Ligands

Carla Bazzicalupi, Craig Grimmer and Igor Vasyl Nikolayenko

Supplementary Information

Contents

Tables

Table S1. Ni(II) complexes reported in literature. … 4

Table S2. Cu(II) complexes reported in literature. … 5

Structure Summary … 6

Table S3. NMR coupling constants derived for the tetraphenylphosphonium cation in DMSO-d_6 solvent at 30 °C. … 7

Table S4. Selected values of the excited state parameters computed for the YNC core. … 8

Table S5. Computed NBOs and their parameters for the YNC core. … 8

Table S6. Selected values of the excited state parameters computed for RNC. … 9

Table S7. Computed NBOs and their parameters for RNC. … 9

Table S8. Comparison of the selected bond lengths (Å) and valence angles (°) for the experimental (XRD, (**6**) and computed structure of the YNC core. … 10

Table S9. Comparison of the selected bond lengths (Å) and valence angles (°) for the experimental (XRD, (**7**)) and computed structure of RNC. … 10

Schemes

Scheme S1. A segment of tetraphenylphosphonium cation with the labelling scheme used for the assignment of NMR coupling constants in this compound. … 11

Figures

Figure S1. FTIR spectrum of **(1)** ≡ $[\{Cu(II)L1H_{-1}\}^{+}(OH_2)]_2^{2+} \cdot 2NO_3^{-} \cdot H_2O$ … 12

Figure S2. FTIR spectrum of **(2)** ≡ $[\{Cu(II)L3H_{-1}\}^{+}(OH_2)]_2^{2+} \cdot 2BF_4^{-} \cdot 2H_2O$. … 12

Figure S3. FTIR spectrum of **(5)** ≡ $[\{Li(OH_2)_3\}^{+}\{Cu(II)L2H_{-3}\}^{-}]^{0} \cdot H_2O$. … 13

Figure S4. MS-ToF spectrum of **(5)**. … 13

Figure S5. FTIR spectra of **(6)** ≡ $[PPh_4]^{+} \cdot [Ni(II)L2H_{-3}]^{-} \cdot H_2O$. **Top**: Spectrum 100. **Bottom**: Alpha II Platinum. … 14

Figure S6. ^{1}H NMR spectrum of **(6)**. … 15

Figure S7. A fragment of ^{1}H NMR spectrum for YNC representing resonances attributable to the tetraphenylphosphonium (TPP) cation. … 15

Figure S8. GCOSY NMR spectrum of **(6)**. … 16

Figure S9. ^{13}C-{^{1}H} NMR spectrum of **(6)**. … 16

Figure S10. ^{13}C-{^{1}H} NMR spectrum of the TPP cation in **(6)**. … 17

Figure S11. DEPT-135 NMR spectrum of **(6)**. … 17

Figure S12. GHSQC NMR spectrum of **(6)**. … 18

Figure S13. GHMBC NMR spectrum of **(6)**. … 18

Figure S14. ^{15}N projection of GHMBC NMR spectrum of **(6)**. … 19

Figure S15. GHMBC-^{15}N NMR spectrum of **(6)**. … 15

Figure S16. ^{31}P NMR spectrum of **(6)**. … 20

Figure S17. Experimental (top) and simulated (bottom) ^{1}H spectrum of the tetraphenylphosphonium (TPP) cation. … 20

Figure S18. MS-ToF spectrum of **(6)**. … 21

Figure S19. FTIR spectra of **(7)** ≡ $[Ni(II)L2'H_{-2}]^0$. … 22

Figure S20. FTIR spectra of **(8)** ≡ $[Ni(II)L2'H_{-2}]^0$. … 23

Figure S21. ^{1}H NMR spectrum of **(7)**. … 24

Figure S22. GCOSY NMR spectrum of **(7)**. … 24

Figure S23. ^{13}C-{^{1}H} NMR spectrum of **(7)**. … 25

Figure S24. GHSQC NMR spectrum of **(7)**. … 25

Figure S25. GHMBC NMR spectrum of **(7)**. … 26

Figure S26. ^{15}N projection of GHMBC NMR spectrum of **(7)**. … 26

Figure S27. GHMBC-^{15}N NMR spectrum of **(7)**. … 27

Figure S28. MS-ToF spectrum of **(7)**. … 28

Figure S29. Computed NBOs involved in the electron transitions that account for the UV-Vis spectrum of YNC (left) and RNC (right). … 29

Figure S30. Computed energy diagram for YNC (left) and RNC (right). Bonding and antibonding states are separated by the dashed line. … 30

Synthesis and Characterisation

Synthesis S1. **(1)** = $[\{Cu(II)L1H_{-1}\}^{+}(OH_2)]_2^{2+} \cdot 2NO_3^{-} \cdot H_2O$ … 31

Synthesis S2. **(2)** = $[\{Cu(II)L3H_{-1}\}^{+}(OH_2)]_2^{2+} \cdot 2BF_4^{-} \cdot 2H_2O$ … 31

Synthesis S3. **(3)** = $[\{Cu(II)L3H_{-1}\}^{+}Cl^{-}]_2^{0} \cdot 4H_2O$ … 31

Synthesis S4. **(4)** = $[\{Cu(II)L3H_{-1}\}^{+}Cl^{-}]_2^{0} \cdot L3 \cdot 2H_2O$ … 31

Synthesis S5. **(5)** = $[\{Li(OH_2)_3\}^{+}\{Cu(II)L2H_{-3}\}^{-}]^{0} \cdot H_2O$ … 32

Synthesis S6. **(6)** = $[PPh_4]^{+} \cdot [Ni(II)L2H_{-3}]^{-} \cdot H_2O$ … 32

Synthesis S7-S8. **(7)–(8)** = $[Ni(II)L2'H_{-2}]^0$ … 33

Structure Refinement

SR S1. **(1)** = $[\{Cu(II)L1H_{-1}\}^{+}(OH_2)]_2^{2+} \cdot 2NO_3^{-} \cdot H_2O$ … 34

SR S2. **(2)** = $[\{Cu(II)L3H_{-1}\}^{+}(OH_2)]_2^{2+} \cdot 2BF_4^{-} \cdot 2H_2O$ … 34

SR S3. **(3)** = $[\{Cu(II)L3H_{-1}\}^{+}Cl^{-}]_2^{0} \cdot 4H_2O$ … 35

SR S4. **(4)** = $[\{Cu(II)L3H_{-1}\}^{+}Cl^{-}]_2^{0} \cdot L3 \cdot 2H_2O$ … 35

SR S5. **(5)** = $[\{Li(OH_2)_3\}^{+}\{Cu(II)L2H_{-3}\}^{-}]^{0} \cdot H_2O$ … 35

SR S7. **(7)** = $[Ni(II)L2'H_{-2}]^0$ … 35

SR S8. **(8)** = $[Ni(II)L2'H_{-2}]^0$ … 35

Tables

Table S1. Ni(II) complexes reported in literature (numbers in square brackets are references in the main text).

**Anionic complexes: 2($N_{ox}N_{ad}$) coordination mode**		
**L1**	**L2**	**L3**
$\{[Ni(II)L1H_{-3}]^- \cdot [Li(OH_2)_2]^+\}_2^0 \cdot H_2O$ [8] JOCJUG[1] a neutral dimer of two anionic sppm[2] complexes bridged by two hydrated lithium cations	$[Ni(II)L2H_{-3}]^- \cdot \frac{1}{2}[Ni(II)(OH_2)_6]^{2+}$ [9] NOXBOR sppm	$[Ni(II)L3H_{-3}]^- \cdot [AsPh_4]^+ \cdot H_2O$ [14] XAPHEC sppm
	$[Ni(II)L2H_{-3}]^- \cdot \frac{1}{2}[Ni(II)(OH_2)_2]^{2+} \cdot 2pn \cdot 2H_2O$ [13] BICFUP sppm	
	$[Ni(II)L2H_{-3}]^- \cdot [Ni(II)(im)_4(OH_2)_2]^{2+}$ [13] BICGAW sppm	
	$[Ni(II)L2H_{-3}]^- \cdot [PPh_4]^+ \cdot H_2O$ [14] XAPHAY, XAPHAY01 sppm	

[1] The compounds with structures determined by XRD, with their CSD codes [39] and structural motifs.
[2] Sppm stands for *square-planar pseudo-macrocycle*.

Table S2. Cu(II) complexes reported in literature (numbers in square brackets are references in the main text).

Cationic complexes: $2(N_{ox}O_{ad})$ coordination mode		
**L1**	**L2**	**L3**
$[\{Cu(II)L1H_{-1}\}^{+}(OH_2)]_2^{2+} \cdot 2ClO_4^{-}$ [8]	$[Cu(II)L2H_{-1}]_2^{2+} \cdot 2NO_3^{-}$ [9]	$[\{Cu(II)L3H_{-1}\}^{+}(OH_2)]_2^{2+} \cdot 2ClO_4^{-} \cdot 2H_2O$ [12] NUFSUC, NUFSUC1 sp-tpp
$[\{Cu(II)L1H_{-1}\}^{+}(MeOH)]_2^{2+} \cdot 2ClO_4^{-} \cdot MeOH$ [17] KAGGUW sp-tpp[3]	$[\{Cu(II)L2H_{-1}\}^{+}(ClO_4^{-})(OH_2)]_n^{0}$ [17] KAGHEH en[5]	
$[\{Cu(II)L1H_{-1}\}^{+}(NO_3^{-})(DMF)]_2^{0} \cdot (CH_3)_2CO$ [17] KAGHAD doh-tpp[4]	$[\{Cu(II)L2H_{-1}\}^{+}(NO_3^{-})_{\frac{1}{2}}(MeOH)]_2^{0} \cdot CHCl_3$ [17] KAGHIL vsp[6]	
**Anionic complexes: $2(N_{ox}N_{ad})$ coordination mode**		
	**L2**	
$[Cu(II)L2H_{-2}]^{0} \cdot H_2O$ [9]	$[Cu(II)L2H_{-3}]^{-} \cdot [Li(OH_2)_4]^{+} \cdot 2H_2O$ [14]	$[Cu(II)L2H_{-3}]^{-} \cdot [PPh_4]^{+} \cdot 4\frac{1}{2}H_2O$ [16] XEQBIF sppm
$[Cu(II)L2H_{-3}]^{-} \cdot [Li(OH_2)_4]^{+} \cdot 2H_2O$ [9] NOXBIL sppm	$[Cu(II)L2H_{-3}]^{-} \cdot [PPh_4]^{+} \cdot 4\frac{1}{2}H_2O$ [14]	
$[Cu(II)L2H_{-3}]^{-} \cdot [Ni(II)(phen)_3]^{2+} \cdot NO_3^{-} \cdot 8H_2O$ [15] XANBET sppm	$[\{Cu(II)L2H_{-4}\}^{2-}\{Co(III)tren\}^{3+}]^{+} \cdot NO_3^{-} \cdot \frac{1}{4}LiNO_3 \cdot 6\frac{1}{4}H_2O$ [14] XAPHUS spmb[7]	$\left\{ \begin{matrix} [Cu(II)L2H_{-3}]_2^{2-} \cdot \\ [Ca(MeOH)_2(OH_2)_2]^{2+} \end{matrix} \right\}_n^{0}$ [18] DUCNIZ spadn[8]
$[Cu(II)L2H_{-3}]^{-} \cdot \frac{1}{2}[Cu(II)(im)_4(OH_2)_2]^{2+} \cdot H_2O$ [13] BICGEA sppm		

[3] Sp-tpp stands for *square-pyramidal two parallel platforms*.
[4] Doh-tpp stands for *distorted octahedral two parallel platforms*.
[5] En stands for *extended network*; in this case second ligand bites only one of the two Cu(II) ions coordinated by the previous ligand, resulting in extended network structure.
[6] Vsp stands for two *V-shaped platforms*.
[7] Spmb stands for *square-planar mouth bridged*, where $\{Cu(II)L2H_{-4}\}^{2-}$ complex anion is chelated at the oximato mouth (via two oximato oxygens) by the $\{Co(III)tren\}^{3+}$ cation, rendering distorted octahedral coordination on the Co(III) ion.
[8] Spadn stands for *square pyramidal anionic* $[Cu(II)L2H_{-3}]_2^{2-}$ *dimer network* where each Cu(II) ion of a dimer is axially coordinated to the neighbouring ligand oximato oxygen, while Ca(II) ions, coordinated to the amide oxygens in the following fashion $>= O \dashrightarrow Ca^{2+} \dashleftarrow O =<$, are responsible for the formation of extended network.

Structure Summary

A review of crystal structures for Ni(II) and Cu(II) complexes with ligands L1-L3 published prior to this work can be summarised as follows. For the Ni(II) complexes with all three ligands only species with the 2($N_{ox}N_{ad}$) coordination were isolated and no coordination from the axial direction was observed in these compounds. In all cases but one, the anionic complex and bulky counter-cation were electrostatically bound in the lattice. In the remaining case [8], two anionic complexes were coordinated *via* their amide oxygens to two lithium cations, which, in turn, were bridged by two water molecules in a diamond-shaped pattern, thus forming neutral complex dimers. For the Cu(II) complexes with all three ligands species with the 2($N_{ox}O_{ad}$) coordination were isolated. For ligands L1 and L3 only such complexes were isolated. For the ligands with an even number of methylene groups in the bridge, 2 or 4, the dimeric complex structure was that of "two parallel platforms", where two nearly planar pseudo-macrocyclic coordination moieties were parallel to each other but at different levels. For the ligand with an odd number of methylene groups in the bridge, 3, the dimeric complex structure was "V-shaped", where the planes of such moieties were at an angle of 65.5° to each other [17], CSD code: KAGHIL. Yet another complex structure was of a polymeric nature, where two Cu(II) centres were coordinated not by two but by three ligand chelates [17], CSD code: KAGHEH. In all cases of reliably studied complexes of this nature, there was coordination of monodentate ligands from the axial direction, either by one such ligand (H_2O, $MeOH$, Cl^-) or by a pair of ligands ({DMF, NO_3^-}, {H_2O, ClO_4^-}, {$MeOH$, NO_3^-}), rendering respectively a square-pyramidal or a distorted octahedral coordination of the Cu(II) centre. Numerous complexes with the 2($N_{ox}N_{ad}$) coordination were isolated but only for ligand L2[1]. In the majority of cases the anionic complex $[Cu(II)L2H_{-3}]^-$ and a bulky counter-cation were only electrostatically bound in the lattice. In one case [14], CSD code: XAPHUS, the fully deprotonated oximato mouth of the anionic complex was bridged by another cation, $\{Co(III)tren\}^{3+}$. In yet another case [16], CSD code: DUCNIZ, each Cu(II) ion was found in square-pyramidal environment, due to axial coordination to the neighbouring ligand oximato oxygen, affording anionic $[Cu(II)L2H_{-3}]_2^{2-}$ dimers. The latter, in turn, were stitched into an extended network by Ca(II) ions coordinated to amide oxygen atoms.

[1] We attribute this phenomenon to higher thermodynamic stability of the six-member coordination ring. In this case the conformation of polymethylene link resembles the "flap of an envelope" and minimises mutual repulsion of the methylene protons in addition to optimal angles of the coordination bonds. For the five- and seven-member coordination rings both of these conditions are not met and the stability of resulting complexes is reduced to the point that they were not isolated in the solid state.

Table S3. NMR coupling constants derived for the tetraphenylphosphonium cation in DMSO-d_6 solvent at 30 °C.

**^{31}P – ^{13}C coupling constants**		
H_A = ^{1}H at 7.97 ppm	H_B = $H_{B'}$ = ^{1}H at 7.82 ppm	H_C = $H_{C'}$ = ^{1}H at 7.74 ppm
^{13}C at 135.3 ppm from HSQC	^{13}C at 130.4 ppm from HSQC	^{13}C at 134.5 ppm from HSQC
J_{PC} = 3.0 Hz from ^{13}C	J_{PC} = 12.8 Hz from ^{13}C	J_{PC} = 10.5 Hz from ^{13}C
**^{1}H – ^{1}H and ^{31}P – ^{1}H coupling constants**		
Spins	Estimated J_{HH} /Hz	Estimated J_{PH} /Hz
CC′	1.3	
CB = C′B′	8.0	
CB′=C′B	0.7	
CA = C′A	1.1	
BB′	1.3	
AB = AB′	7.3	
PA = PA′		12.9
PB = PB′		3.7
PC		1.9

Table S4. Selected values of the excited state parameters computed for the YNC core.

Calculation type	Calculation method	Basis set	Charge	Spin	Computed energy /au	Computed dipole moment /Debye
SP[1]	RB3LYP	6-311++G(3d,2p)	-1	Singlet	-2379.38975056	1.8852
**Excited state**	**$\Delta\varepsilon$ /eV**[2]	**λ /nm**[3]	***F***[4]	**Transition orbitals**		**Orbital coefficient**
n = 6	3.4827	356.00	0.1128	77 → 79		0.6589
				78 → 79		-0.2094
n = 11	4.1008	302.34	0.0936	74 → 79		-0.1676
				75 → 79		0.6105
				76 → 81		-0.1031
				78 → 81		0.2240

[1] *Single point* (energy) calculation.
[2] Transition energy.
[3] Wavelength of the electron transition.
[4] Oscillator strength.

Table S5. Computed NBOs and their parameters for the YNC core.

Orbital number	Occupancy	Orbital type	Centered on	Approximate character
74	1.8531	LP[1]	O2	p_x^2
75	1.9885	LP	O3	p_x^2
76	1.9756	LP	Ni	$d_{z^2}^2$
77	1.9730	LP	N2	p_z^2
78 (HOMO)	1.9399	LP	N3	p_z^2
79 (LUMO)	0.0640	RY*[2]	N1–C2	π^*
80	0.0096	RY*	N4–C8	π^*
81	0.0045	RY*	C3–O2	π^*

[1] *Lone pair*.
[2] *Rydberg* antibonding orbital.

Table S6. Selected values of the excited state parameters computed for RNC.

Calculation type	Calculation method	Basis set	Charge	Spin	Computed energy /au	Computed dipole moment /Debye
SP	RB3LYP	6-311++G(3d,2p)	0	Singlet	-2377.51833026	2.3957
**Excited state**	**Δε /eV**	**λ /nm**	***f***	**Transition orbitals**	**Orbital coefficient**	
n = 2	2.5774	492.51	0.0204	73 → 79	-0.1280	
				74 → 79	0.6165	
				76 → 77	0.3192	
n = 3	2.5383	488.46	0.0341	74 → 77	-0.3226	
				76 → 77	0.6154	
n = 6	3.1593	392.44	0.0114	72 → 77	0.1577	
				75 → 77	0.4831	
				76 → 78	-0.4706	
n = 11	3.8284	323.85	0.0384	72 → 77	0.3740	
				72 → 78	0.1656	
				75 → 78	0.5555	

Table S7. Computed NBOs and their parameters for RNC.

Orbital number	Occupancy	Orbital type	Centered on	Approximate character
72	1.8153	LP	O2	p_x^2
73	1.9879	LP	Ni	d_{xz}^2
74	1.9764	LP	Ni	d_{yz}^2
75	1.9709	LP	Ni	$d_{z^2}^2$
76 (HOMO)	1.9380	BD[1]	C5–H6	π^2
77 (LUMO)	0.0481	RY*	N2–C4	π^*
78	0.0093	RY*	N3–C6	π^*
79	0.0042	RY*	N1–C2	π^*

[1] *Bonding* orbital.

Table S8. Comparison of the selected bond lengths (Å) and valence angles (°) for the experimental (XRD, **(6)** [14]) and computed structure of the YNC core.

XRD				Computed			
Ni–N1	1.879(2)	N2–C4	1.462(3)	Ni–N1	1.903	N2–C4	1.453
Ni–N4	1.872(1)	N3–C5	1.466(2)	Ni–N4	1.900	N3–C5	1.455
Ni–N2	1.865(1)	C4–C9	1.517(3)	Ni–N2	1.898	C4–C9	1.527
Ni–N3	1.869(2)	C5–C9	1.513(3)	Ni–N3	1.883	C5–C9	1.528
O1–N1	1.361(2)			O1–N1	1.354		
O4–N4	1.358(2)	O1–O4	2.431	O4–N4	1.324	O1–O4	2.461
C3–O2	1.250(2)	O1–H1	1.20(4)	C3–O2	1.246	O1–H1	1.067
C6–O3	1.255(3)	O4···H1	1.24	C6–O3	1.248	O4···H1	1.402
N1–C2	1.287(2)			N1–C2	1.283		
N4–C7	1.292(3)	N1–Ni–N2	83.50(7)	N4–C7	1.292	N1–Ni–N2	82.60
C3–N2	1.330(2)	N4–Ni–N3	83.13(7)	C3–N2	1.334	N4–Ni–N3	83.57
C6–N3	1.330(2)	N1–Ni–N4	96.72(7)	C6–N3	1.339	N1–Ni–N4	96.59
C1–C2	1.488(3)	N2–Ni–N3	96.63(7)	C1–C2	1.488	N2–Ni–N3	97.23
C8–C7	1.485(7)	N1–Ni–N3	179.23(7)	C8–C7	1.489	N1–Ni–N3	179.28
C2–C3	1.508(3)	N4–Ni–N2	178.50(7)	C2–C3	1.513	N4–Ni–N2	179.02
C7–C6	1.503(3)	O1–H1···O4	172.9	C7–C6	1.502	O1–H1···O4	170.04

Table S9. Comparison of the selected bond lengths (Å) and valence angles (°) for the experimental (XRD, **(7)**) and computed structure of RNC.

XRD				Computed			
Ni N1	1.877(8)	N2 C4	1.315(14)	Ni–N1	1.905	N2–C4	1.322
Ni N4	1.873(8)	N3 C5	1.325(13)	Ni–N4	1.895	N3–C5	1.321
Ni N2	1.861(7)	C4 C9	1.369(14)	Ni–N2	1.880	C4–C9	1.387
Ni N3	1.851(8)	C5 C9	1.406(12)	Ni–N3	1.864	C5–C9	1.398
O1 N1	1.339(10)	C9 H10	0.930	O1–N1	1.340	C9–H10	1.078
O4 N4	1.342(10)	O1 O4	2.439	O4–N4	1.257	O1–O4	2.486
C3 O2	1.228(11)	O1 H1	1.074	C3–O2	1.216	O1–H1	1.049
C6 O3	1.210(13)	O4 H1	1.415	C6–O3	1.217	O4···H1	1.449
N1 C2	1.282(11)			N1–C2	1.287		
N4 C7	1.274(13)	N1 Ni N2	84.0(3)	N4–C7	1.304	N1–Ni–N2	83.00
C3 N2	1.441(11)	N3 Ni N4	83.7(3)	C3–N2	1.399	N4–Ni–N3	83.93
C6 N3	1.412(11)	N4 Ni N1	97.8(3)	C6–N3	1.417	N1–Ni–N4	97.85
C1–C2	1.488(12)	N2 Ni N3	94.4(4)	C1–C2	1.486	N2–Ni–N3	95.22
C8–C7	1.475(12)	N1 Ni N3	178.2(4)	C8–C7	1.488	N1–Ni–N3	178.33
C2–C3	1.470(16)	N2 Ni N4	178.0(5)	C2–C3	1.498	N4–Ni–N2	179.15
C7–C6	1.509(18)	O1–H1···O4	156.8	C7–C6	1.473	O1–H1···O4	168.35

Schemes

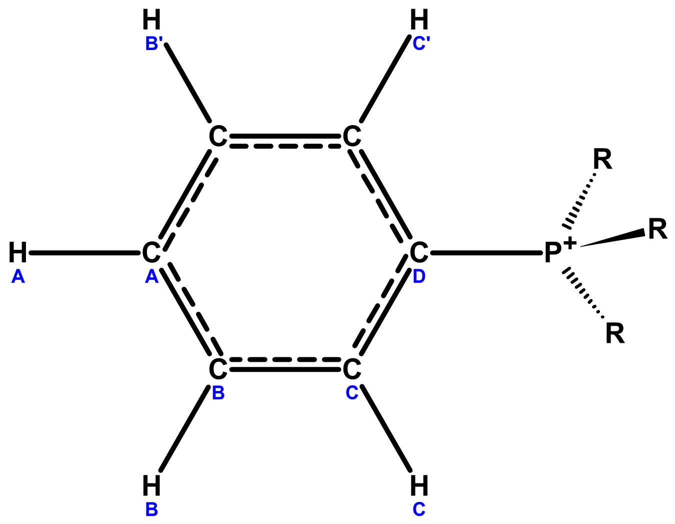

Scheme S1. A segment of tetraphenylphosphonium cation with the labelling scheme used for the assignment of NMR coupling constants in this compound.

Figures

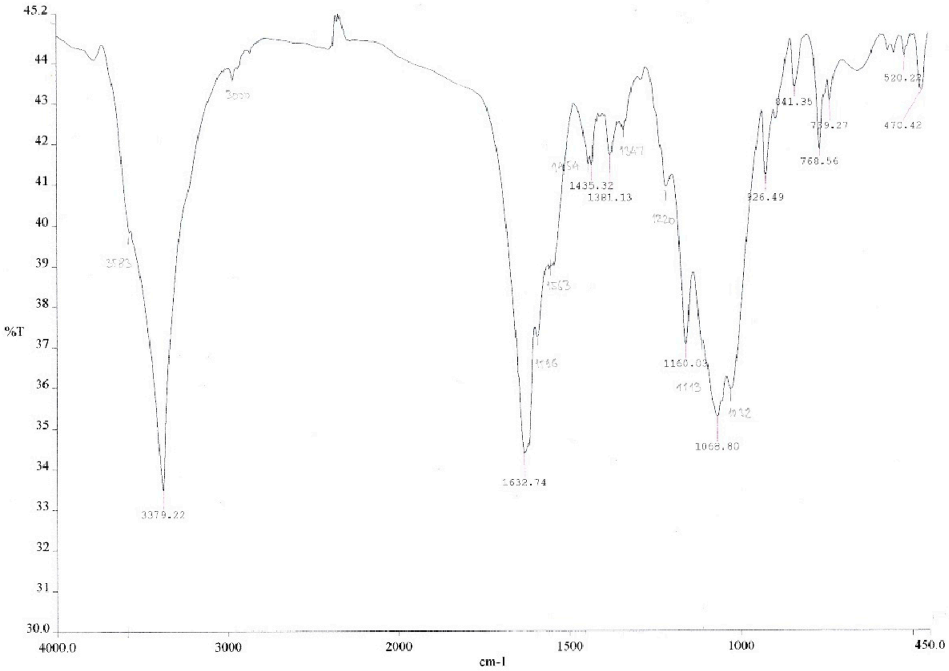

Figure S1. FTIR spectrum of **(1)** ≡ $[\{Cu(II)L1H_{-1}\}^{+}(OH_2)]_2^{2+} \cdot 2NO_3^{-} \cdot H_2O$

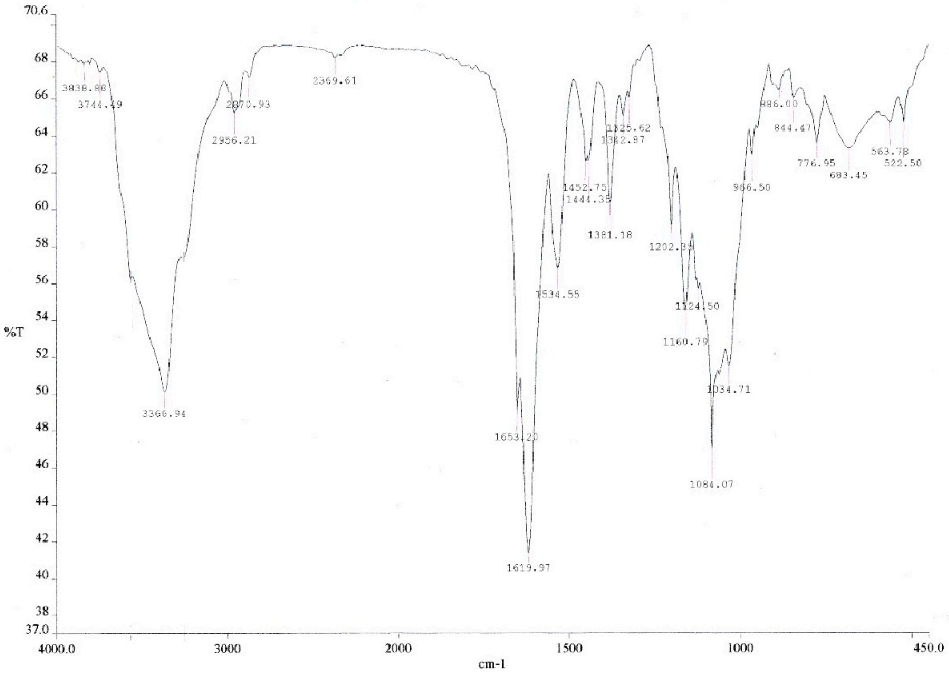

Figure S2. FTIR spectrum of **(2)** ≡ $[\{Cu(II)L3H_{-1}\}^{+}(OH_2)]_2^{2+} \cdot 2BF_4^{-} \cdot 2H_2O$.

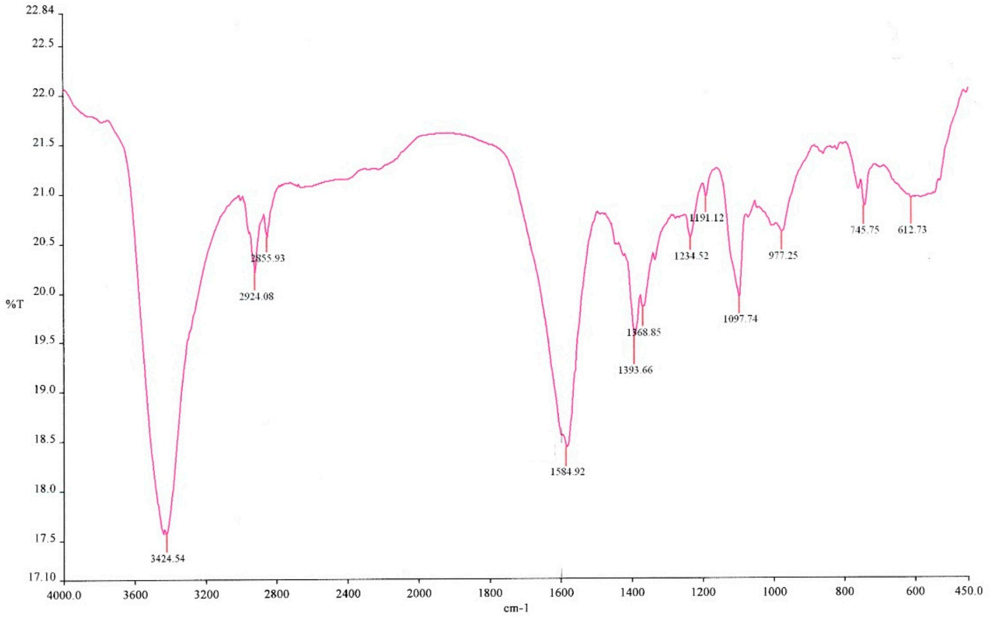

Figure S3. FTIR spectrum of **(5)** ≡ $[\{Li(OH_2)_3\}^{+}\{Cu(II)L2H_{-3}\}^{-}]^{0} \cdot H_2O$.

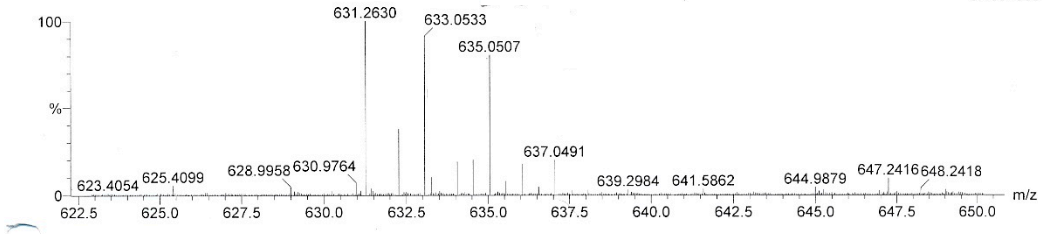

Elemental Composition Report Page 1

Single Mass Analysis
Tolerance = 50.0 PPM / DBE: min = -1.5, max = 50.0
Element prediction: Off
Number of isotope peaks used for i-FIT = 3

Monoisotopic Mass, Even Electron Ions
187 formula(e) evaluated with 6 results within limits (up to 500 best isotopic matches for each mass)
Elements Used:
C: 15-20 H: 25-30 N: 5-10 O: 5-10 Na: 0-1 Cu: 2-5
Igor Nikolayenko
L2_Cu_ppt 46 (0.832) Cm (1:55) TOF MS ES+ 6.48e+003

Minimum:				-1.5			
Maximum:		5.0	50.0	50.0			
Mass	Calc. Mass	mDa	PPM	DBE	i-FIT	i-FIT (Norm)	Formula
633.0533	633.0544	-1.1	-1.7	11.5	376.3	4.1	C20 H27 N8 O8 Cu2
	633.0520	1.3	2.1	8.5	374.3	2.0	C18 H28 N8 O8 Cu2
	633.0504	2.9	4.6	7.5	372.6	0.4	C15 H27 N10 O10 Cu2
	633.0632	-9.9	-15.6	8.5	374.3	2.1	C17 H28 N10 O7 Cu2
	633.0656	-12.3	-19.4	11.5	376.7	4.5	C19 H27 N10 O7 Cu2
	633.0407	12.6	19.9	8.5	375.4	3.2	C19 H28 N6 O9 Na Cu2

Figure S4. MS-ToF spectrum of **(5)**.

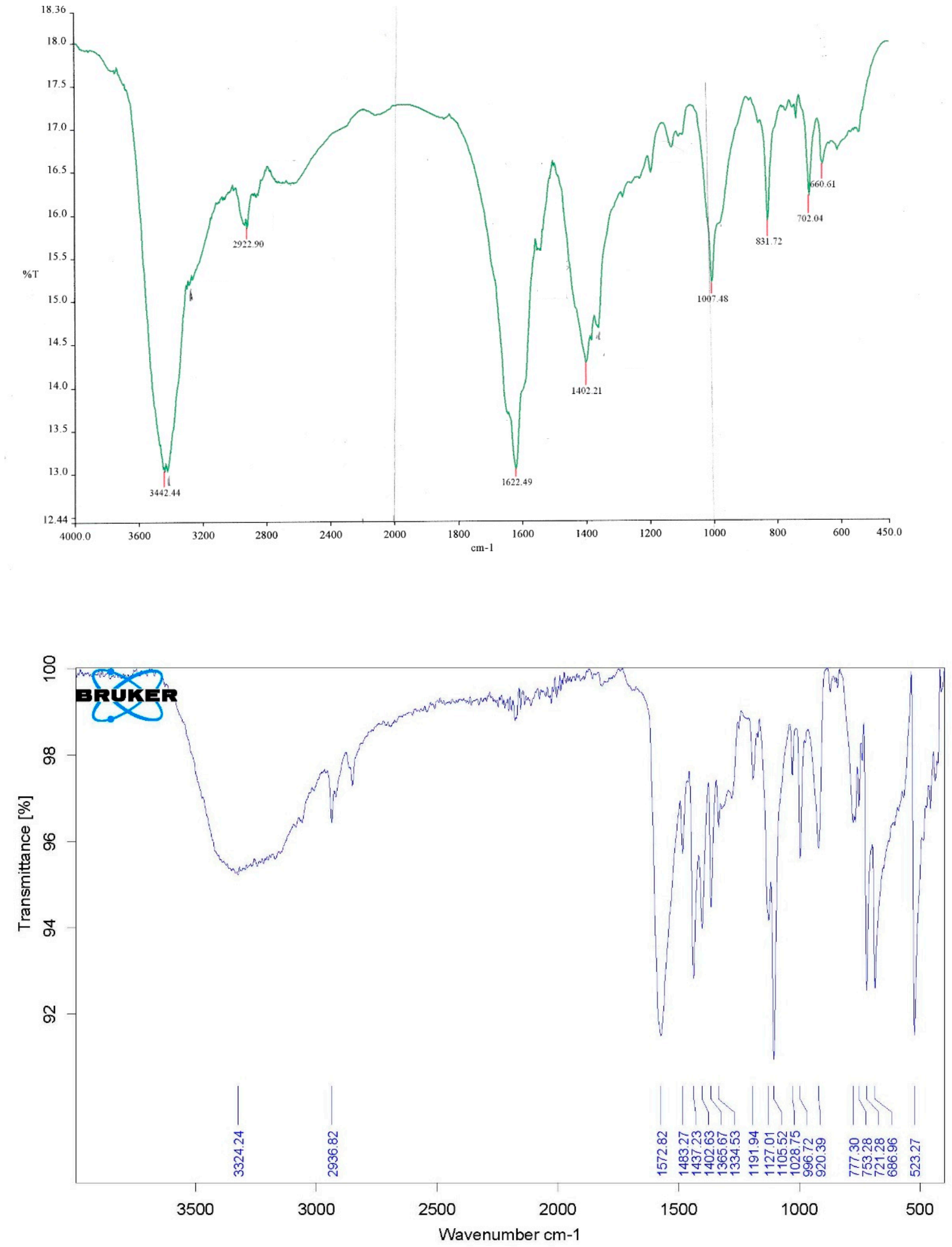

Figure S5. FTIR spectra of **(6)** ≡ $[PPh_4]^+ \cdot [Ni(II)L2H_{-3}]^- \cdot H_2O$. **Top:** Spectrum 100. **Bottom:** Alpha II Platinum.

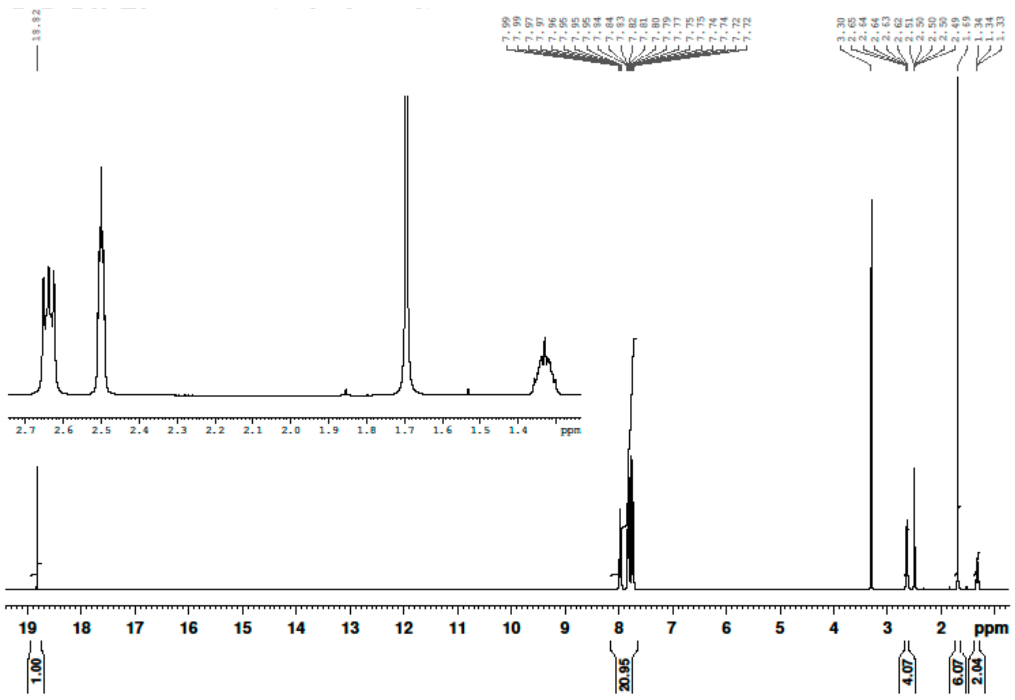

Figure S6. ^{1}H NMR spectrum of **(6)**.

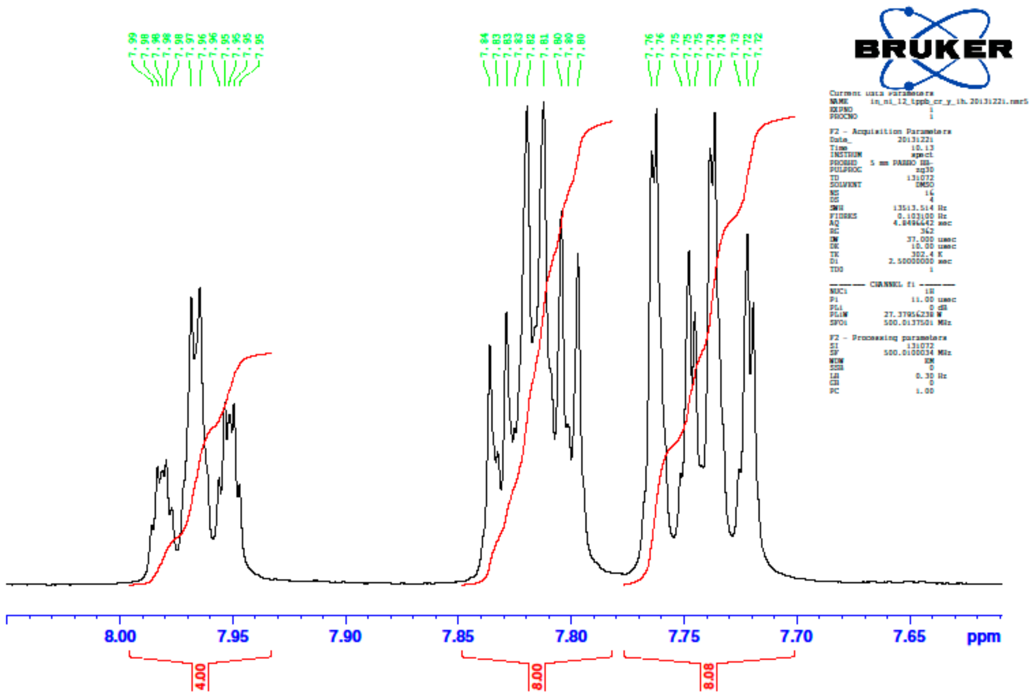

Figure S7. A fragment of ^{1}H NMR spectrum for YNC representing resonances attributable to the tetraphenylphosphonium (TPP) cation.

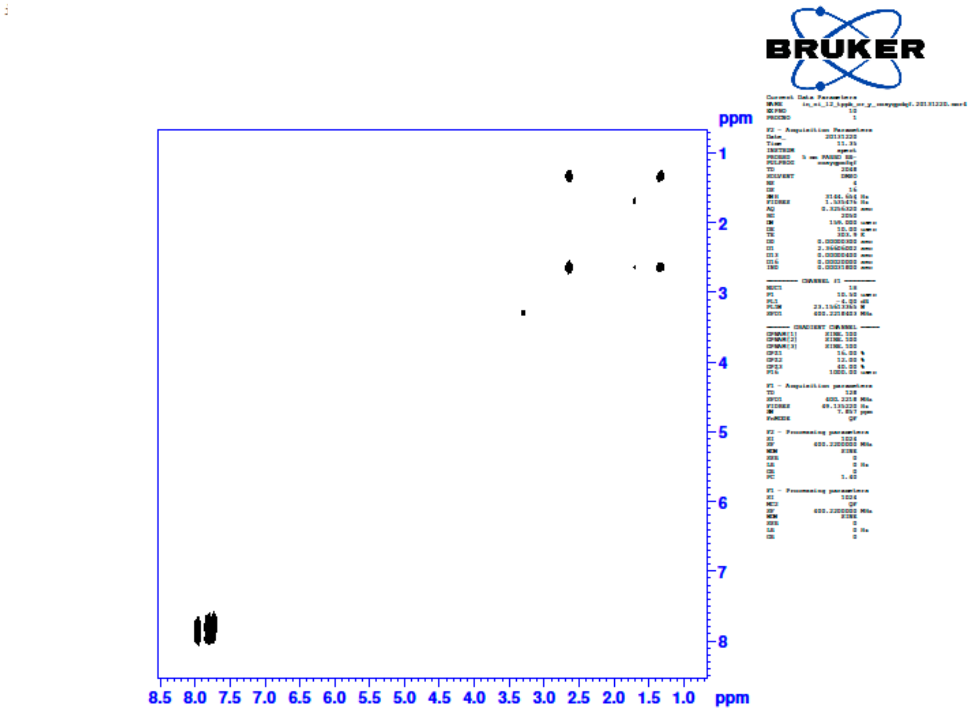

Figure S8. GCOSY NMR spectrum of **(6)**.

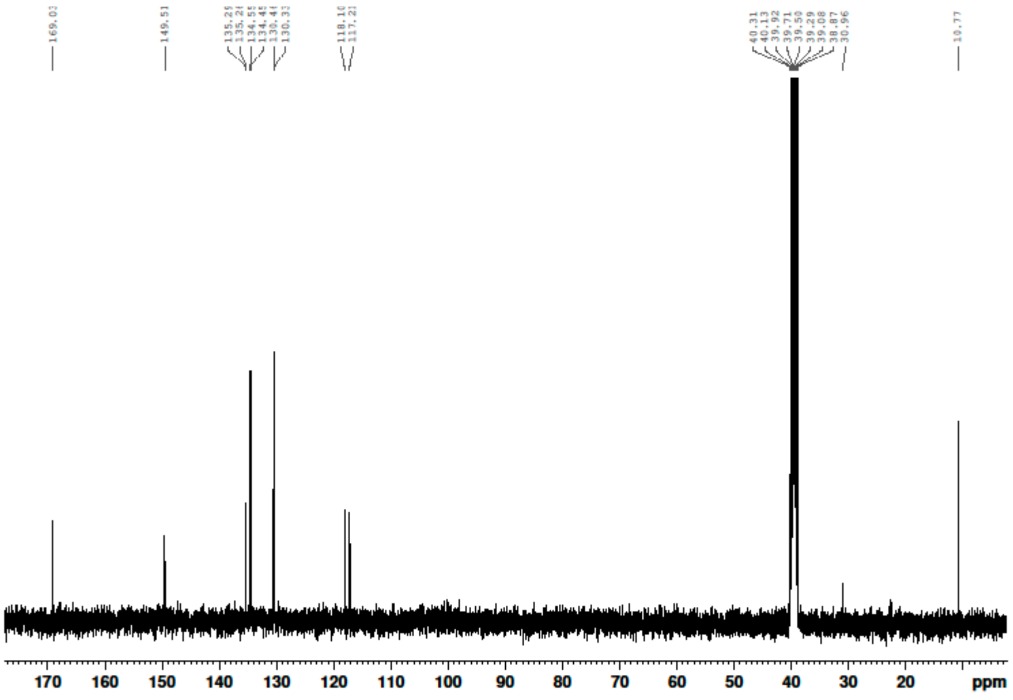

Figure S9. ^{13}C-{^{1}H} NMR spectrum of **(6)**.

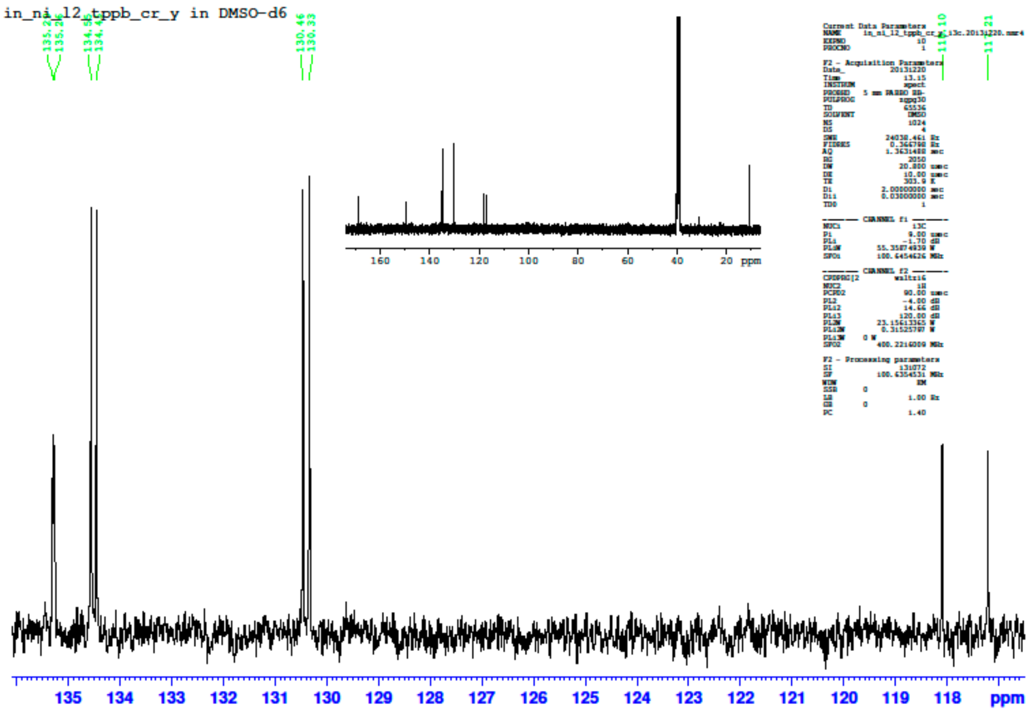

Figure S10. ^{13}C-{^{1}H} NMR spectrum of the TPP cation in **(6)**.

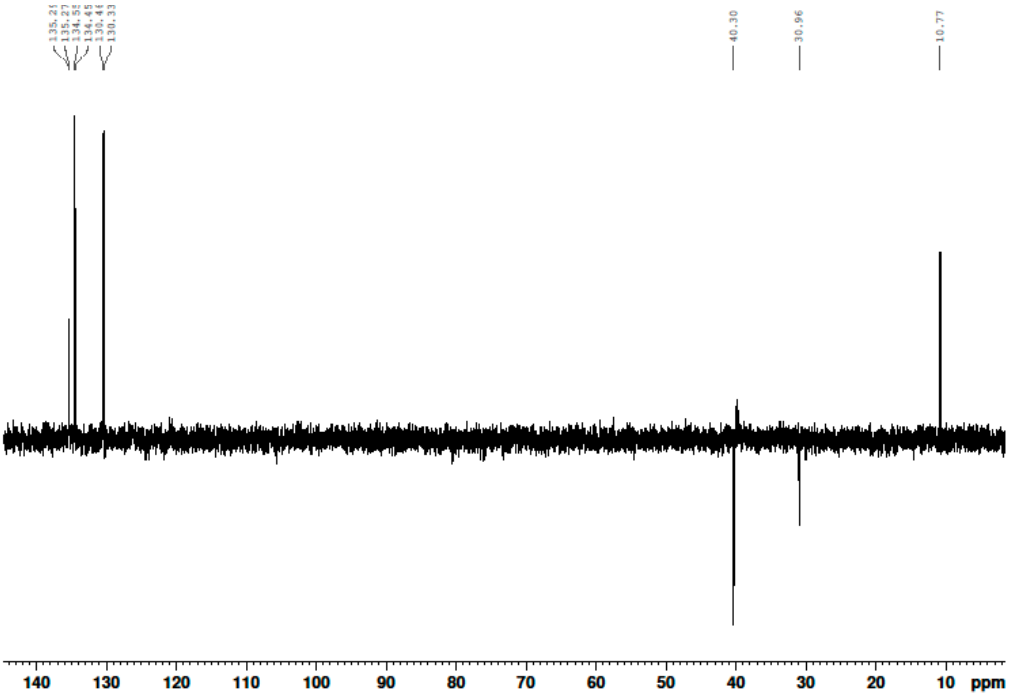

Figure S11. DEPT-135 NMR spectrum of **(6)**.

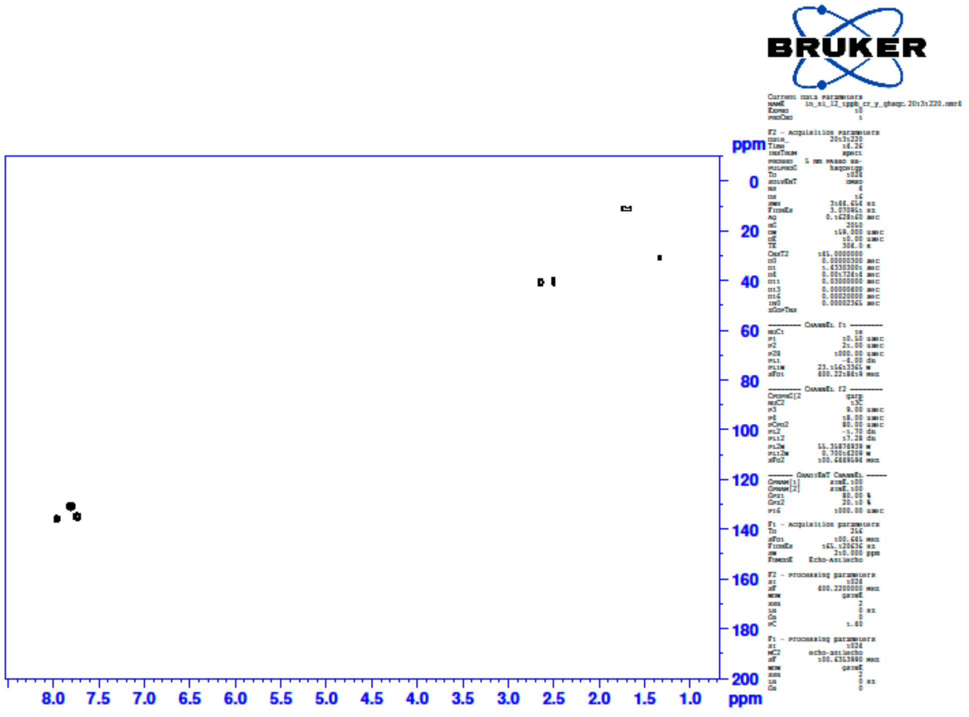

Figure S12. GHSQC NMR spectrum of **(6)**.

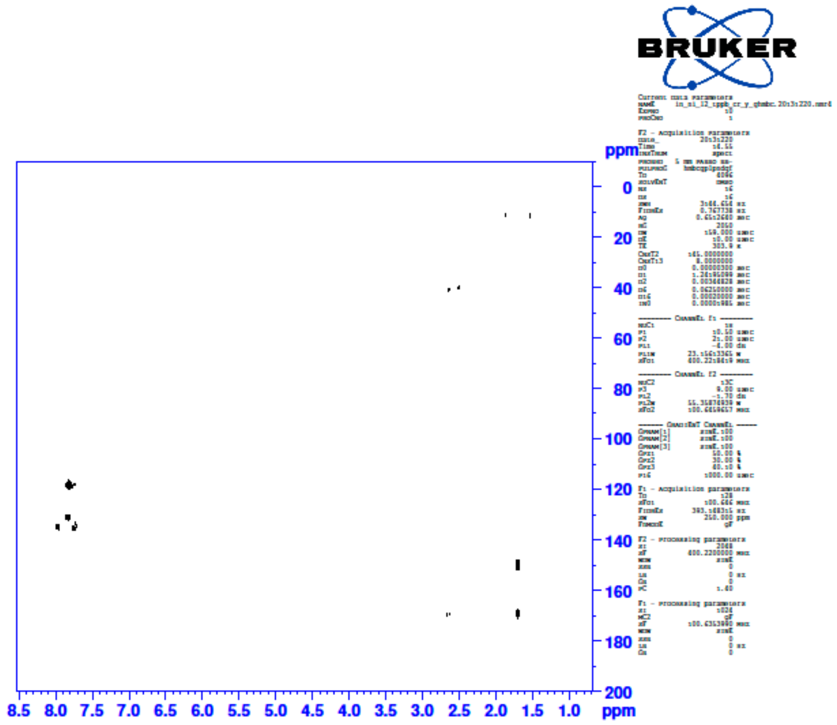

Figure S13. GHMBC NMR spectrum of **(6)**.

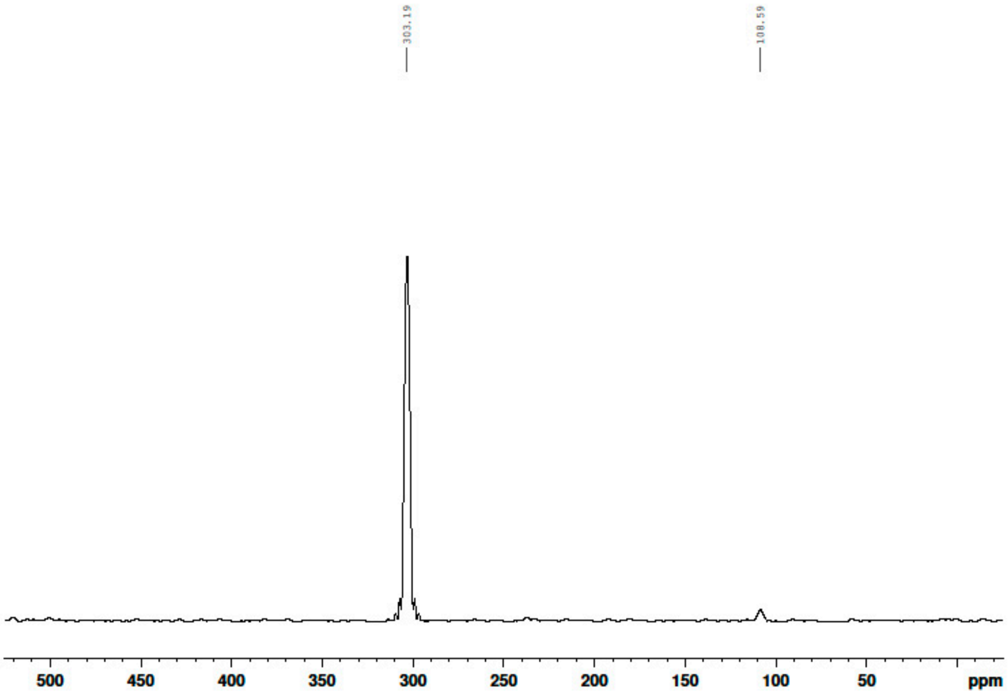

Figure S14. ^{15}N projection of GHMBC NMR spectrum of **(6)**.

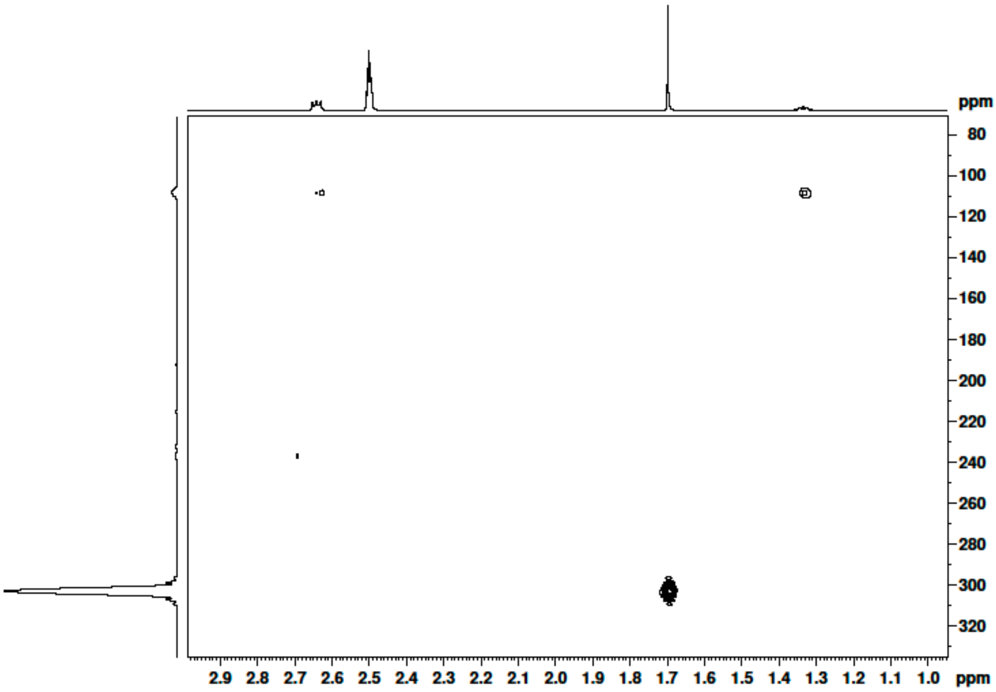

Figure S15. GHMBC-^{15}N NMR spectrum of **(6)**.

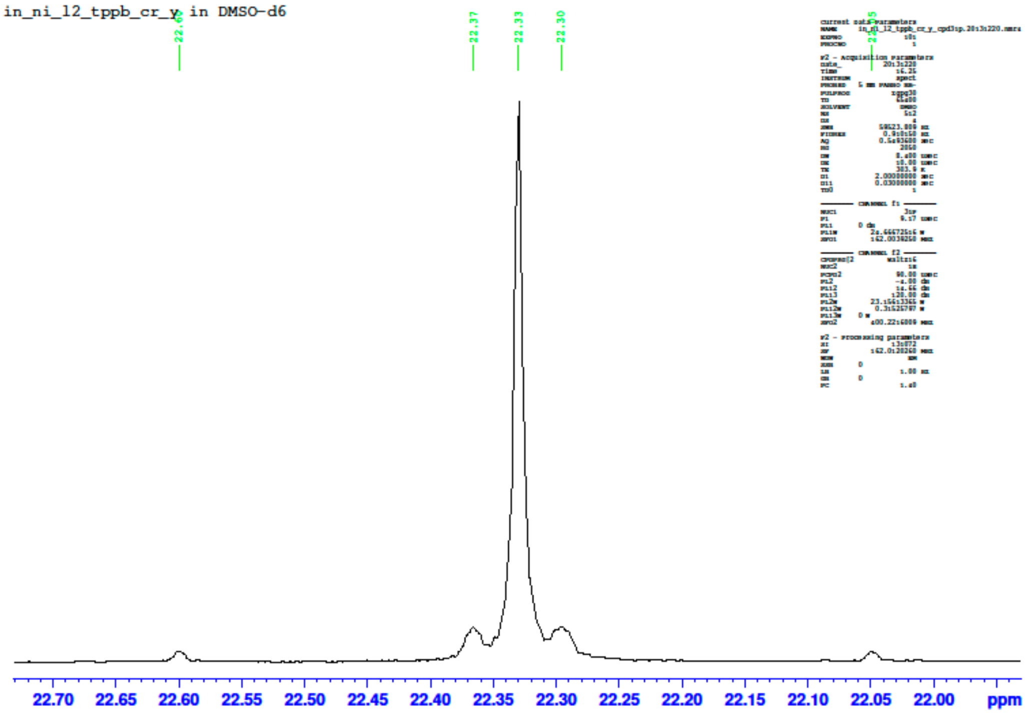

Figure S16. ^{31}P NMR spectrum of **(6)**.

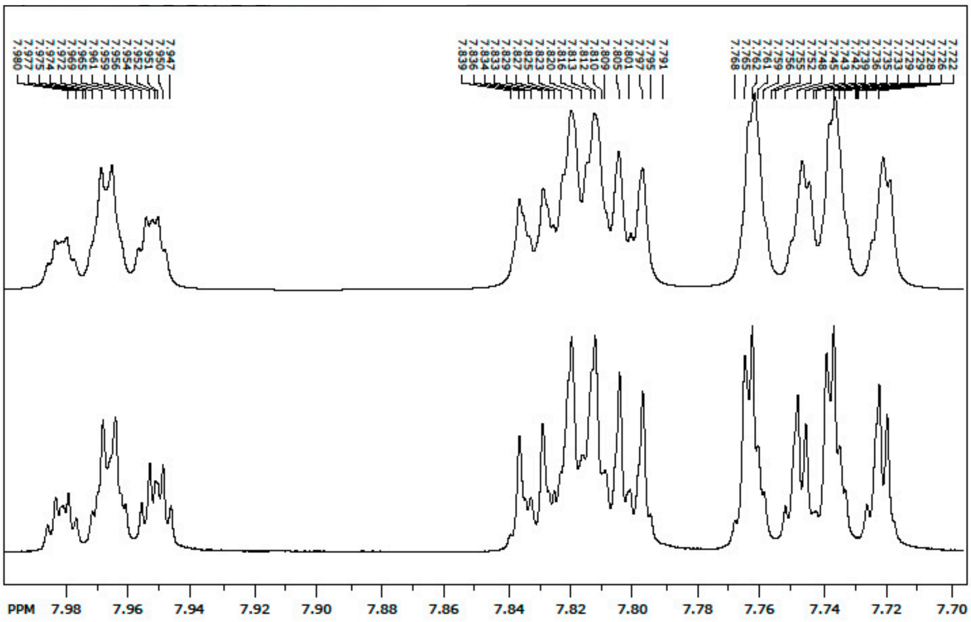

Figure S17. Experimental (top) and simulated (bottom) ^{1}H spectrum of the tetraphenylphosphonium (TPP) cation (simulation performed with SpinWorks-4).

Elemental Composition Report **Page 1**

Single Mass Analysis
Tolerance = 50.0 PPM / DBE: min = -1.5, max = 50.0
Element prediction: Off
Number of isotope peaks used for i-FIT = 3

Monoisotopic Mass, Even Electron Ions
99 formula(e) evaluated with 3 results within limits (up to 500 best isotopic matches for each mass)
Elements Used:
C: 17-25 H: 10-30 N: 8-10 O: 8-10 Na: 0-1 Ni: 1-5
Igor Nikolayenko
L2_Ni_MeOH(red) W+ high cone 10 (0.166) Cm (1:56) TOF MS ES+ 1.67e+004

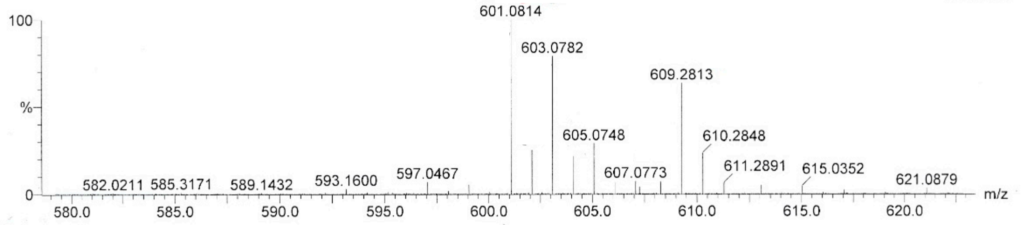

inimum:				-1.5			
Maximum:		5.0	50.0	50.0			
Mass	Calc. Mass	mDa	PPM	DBE	i-FIT	i-FIT (Norm)	Formula
601.0814	601.0815	-0.1	-0.2	9.5	346.8	0.0	C18 H29 N8 O8 Ni2
	601.0816	-0.2	-0.3	16.5	356.0	9.2	C21 H22 N9 O9 Ni
	601.0792	2.2	3.7	13.5	356.0	9.2	C19 H22 N9 O9 Na Ni

Figure S18. MS-ToF spectrum of **(6)**.

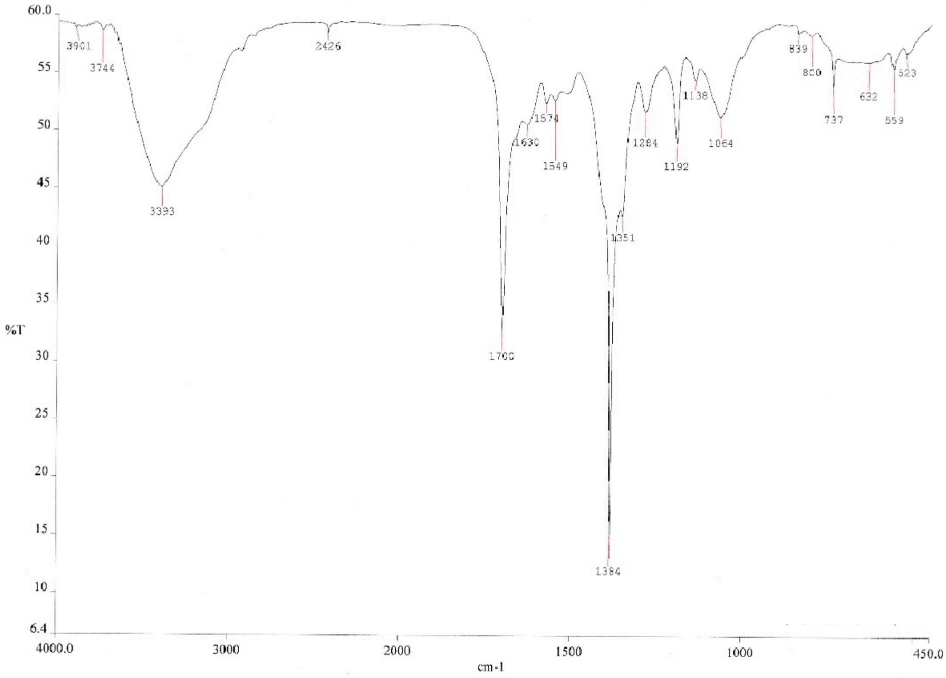

Figure S19. FTIR spectra of **(7)** ≡ $[Ni(II)L2'H_{-2}]^0$

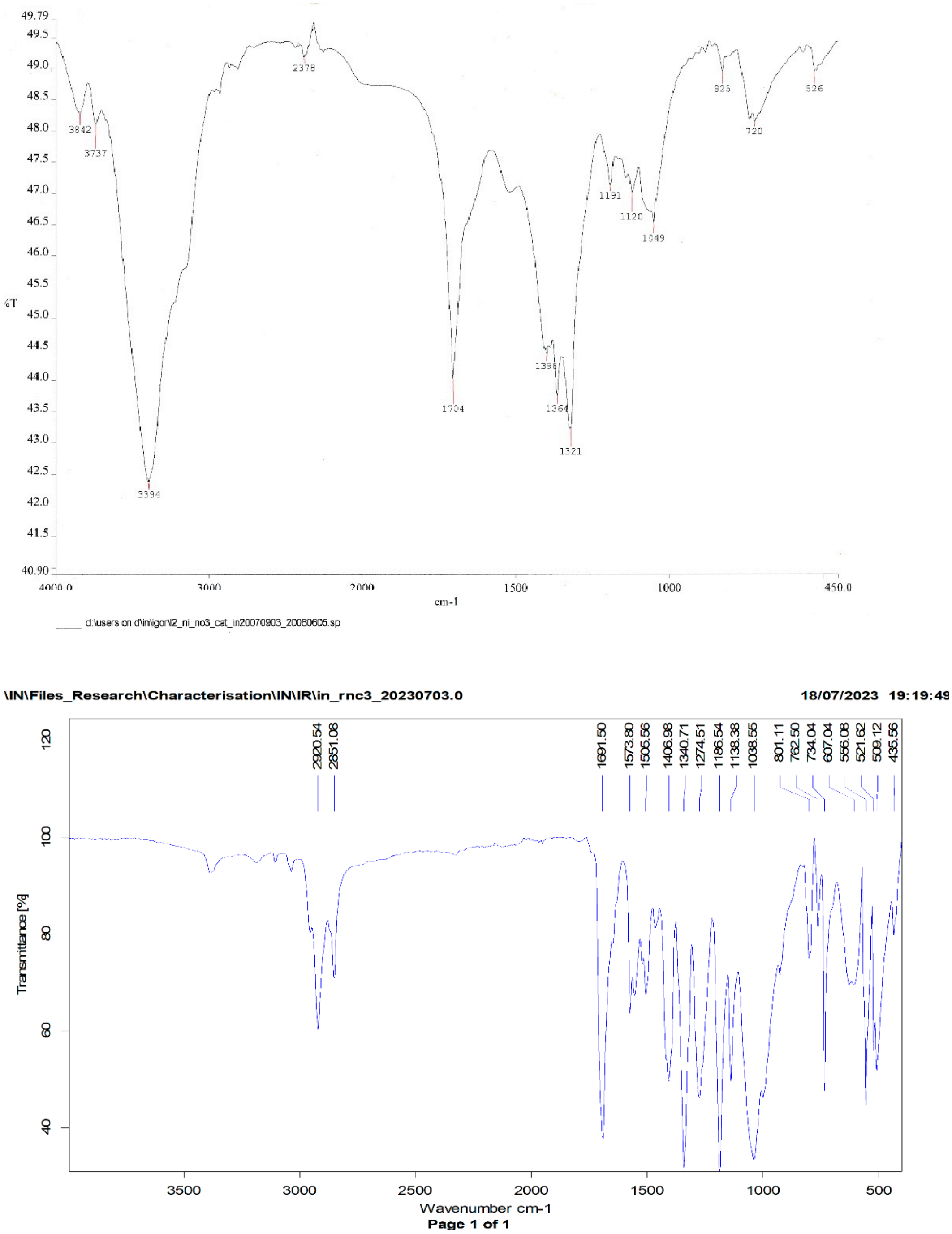

Figure S20. FTIR spectra of **(8)** ≡ $[Ni(II)L2'H_{-2}]^0$. **Top:** Spectrum 100. **Bottom:** Alpha II Platinum.

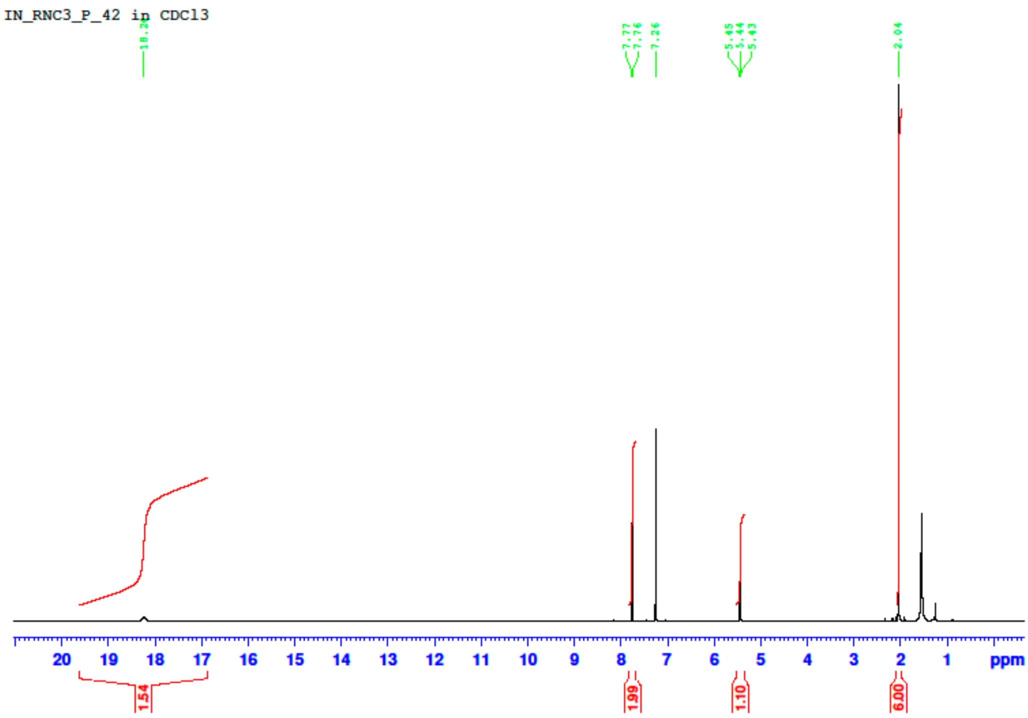

Figure S21. ^{1}H NMR spectrum of **(7)**.

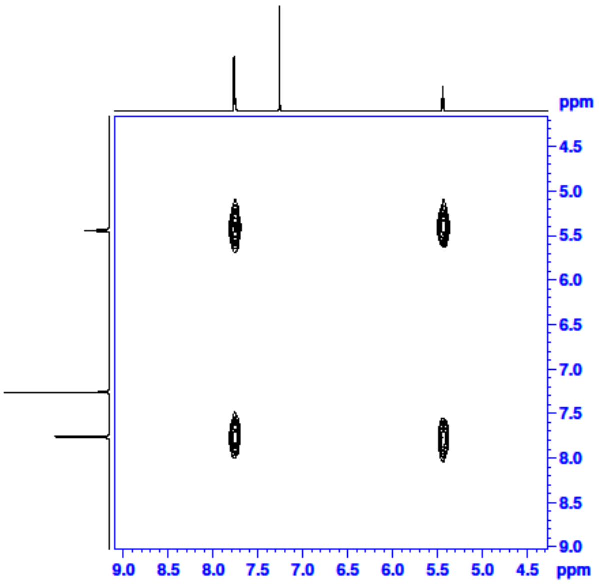

Figure S22. GCOSY NMR spectrum of **(7)**.

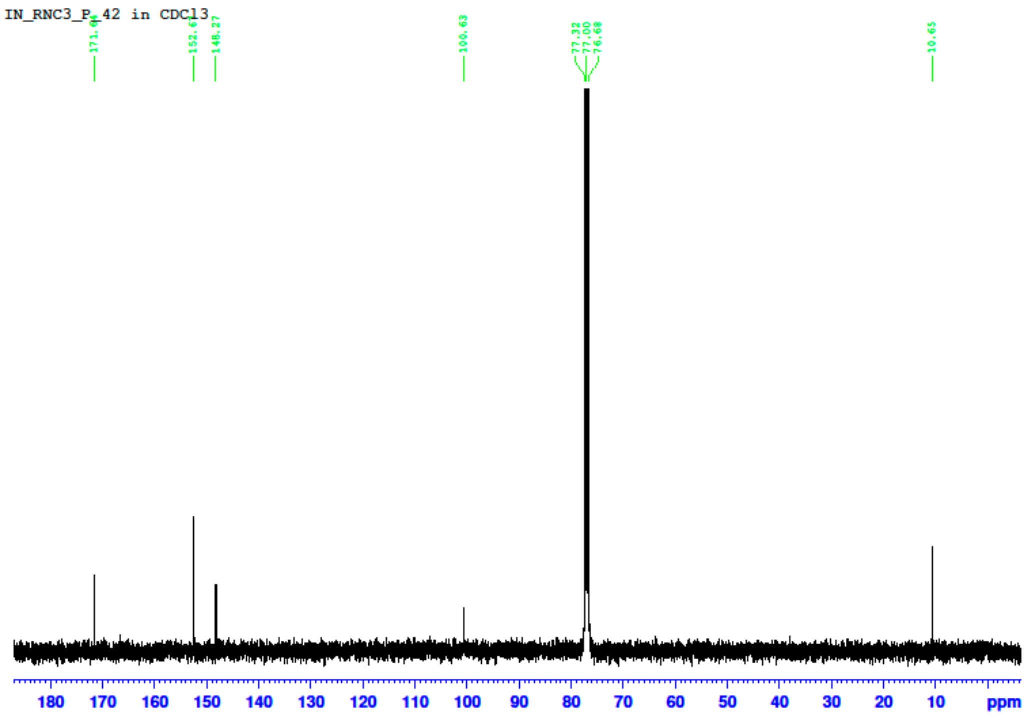

Figure S23. ^{13}C-{^{1}H} NMR spectrum of **(7)**.

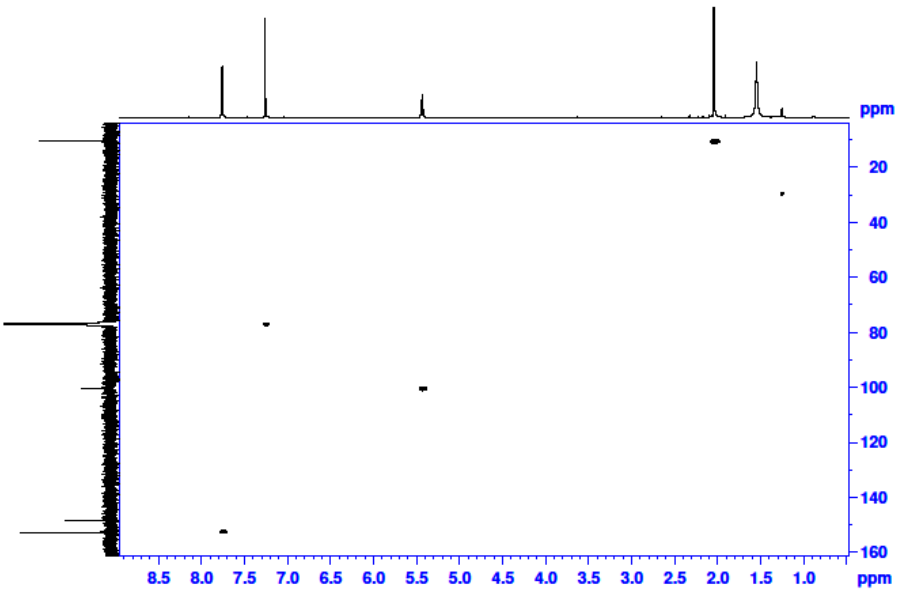

Figure S24. GHSQC NMR spectrum of **(7)**.

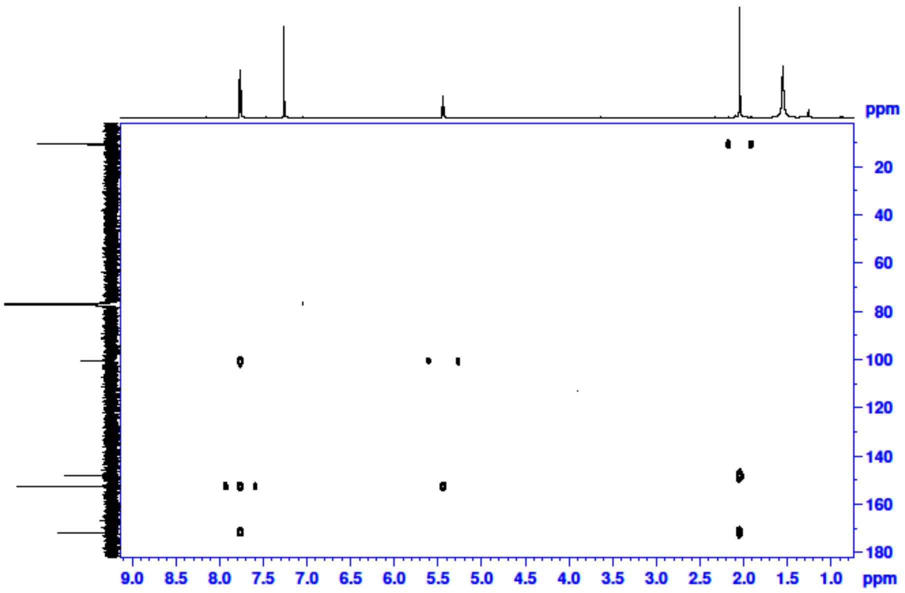

Figure S25. GHMBC NMR spectrum of **(7)**.

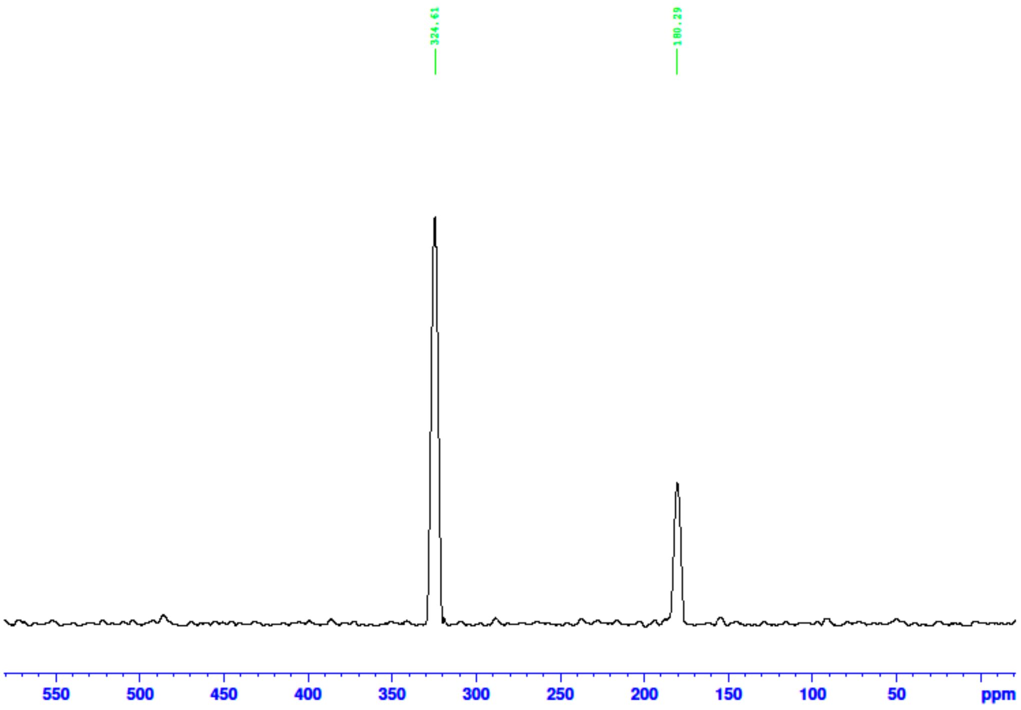

Figure S26. ^{15}N projection of GHMBC NMR spectrum of **(7)**.

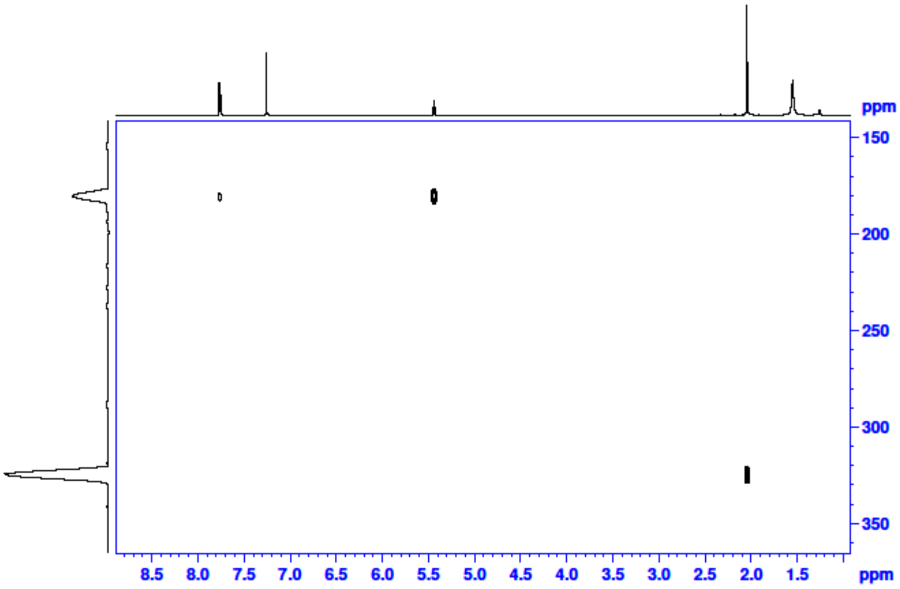

Figure S27. GHMBC-^{15}N NMR spectrum of **(7)**.

Elemental Composition Report **Page 1**

Single Mass Analysis
Tolerance = 50.0 PPM / DBE: min = -1.5, max = 50.0
Element prediction: Off
Number of isotope peaks used for i-FIT = 3

Monoisotopic Mass, Even Electron Ions
662 formula(e) evaluated with 8 results within limits (up to 500 best isotopic matches for each mass)
Elements Used:
C: 10-20 H: 20-30 N: 0-10 O: 0-10 Na: 0-1 Ni: 0-2
Igor Nikolayenko
L2_Ni_stacked 4 (0.056) Cm (1:33) TOF MS ES+
8.24e+003

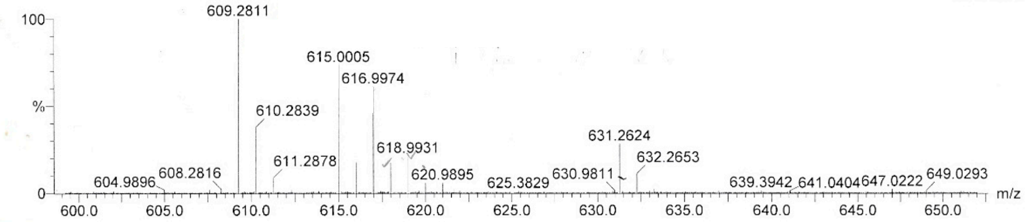

Minimum:				-1.5			
Maximum:		5.0	50.0	50.0			
Mass	Calc. Mass	mDa	PPM	DBE	i-FIT	i-FIT (Norm)	Formula
615.0005	615.0009	-0.4	-0.7	13.5	294.9	1.0	C18 H20 N8 O8 Na Ni2
	614.9969	3.6	5.9	9.5	300.7	6.7	C13 H20 N10 O10 Na Ni2
	614.9896	10.9	17.7	13.5	295.1	1.2	C19 H20 N6 O9 Na Ni2
	615.0121	-11.6	-18.9	13.5	295.8	1.9	C17 H20 N10 O7 Na Ni2
	615.0220	-21.5	-35.0	8.5	300.0	6.1	C15 H24 N8 O10 Na Ni2
	614.9784	22.1	35.9	13.5	296.3	2.4	C20 H20 N4 O10 Na Ni2
	615.0244	-23.9	-38.9	11.5	297.0	3.1	C17 H23 N8 O10 Ni2
	615.0260	-25.5	-41.5	12.5	297.8	3.9	C20 H24 N6 O8 Na Ni2

Figure S28. MS-ToF spectrum of **(7)**.

**YNC**		**RNC**	
Orbital number	Orbital shape	Orbital number	Orbital shape
**81**	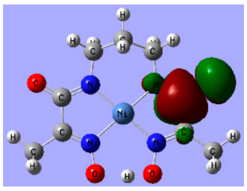	**79**	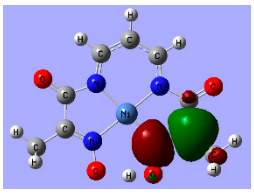
**80**	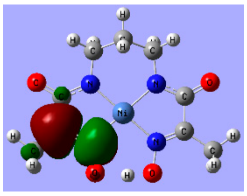	**78**	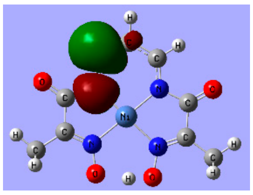
**79**	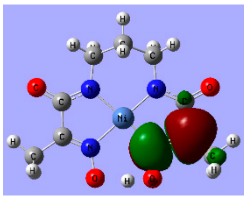	**77**	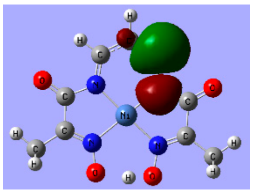
**78**	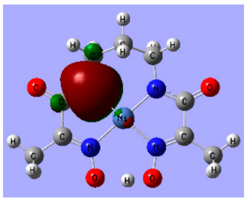	**76**	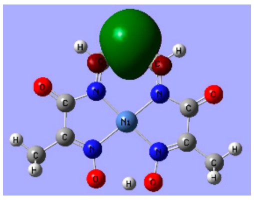
**77**	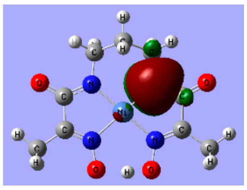	**75**	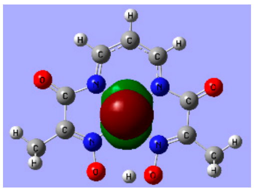
**76**	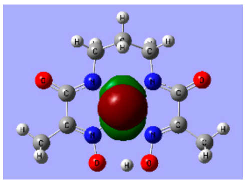	**74**	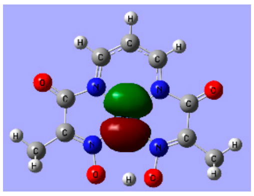
**75**	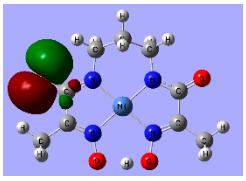	**73**	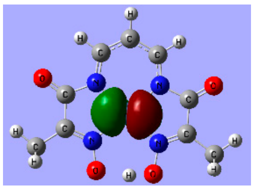
**74**	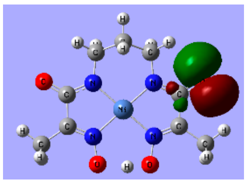	**72**	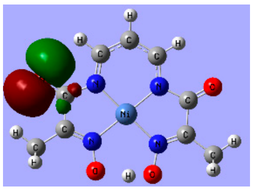

Figure S29. Computed NBOs involved in the electron transitions that account for the UV-Vis spectrum of YNC (left) and RNC (right).

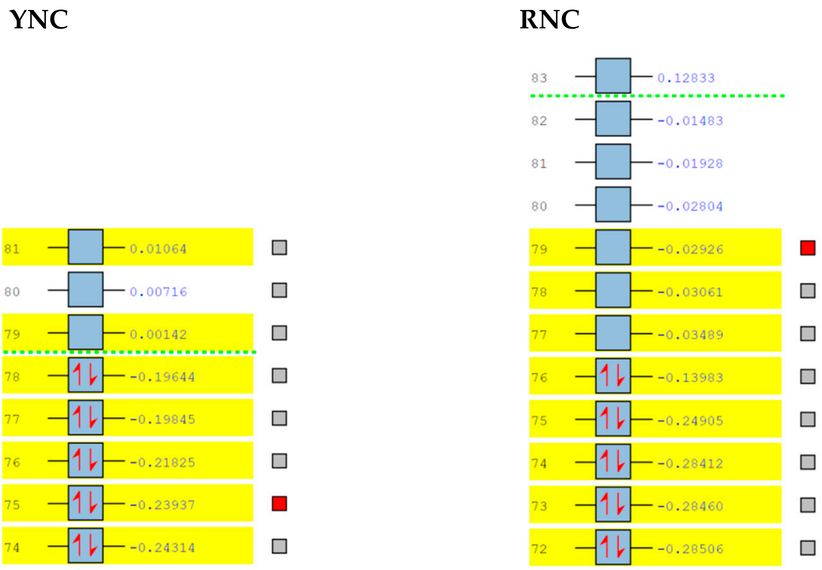

Figure S30. Computed energy diagram for YNC (left) and RNC (right). Bonding and antibonding states are separated by the dashed line.

Synthesis and Characterisation

(1) = $[\{Cu(II)L1H_{-1}\}^{+}(OH_2)]_2^{2+} \cdot 2NO_3^{-} \cdot H_2O$

Synthesis S1.

Ligand L1 (0.2341 g, 1.017 mmol), synthesised as previously reported (Nikolayenko, I.; Barry, J.; Chalmers, C. In Proceedings of the 37th International Conference on Coordination Chemistry, Cape Town, South Africa, 13–18 August 2006; p. 245), was dissolved in ethanol (10 mL) followed by the addition of 0.22 μL of HNO_3(aq, 70 %). $Cu(NO_3)_2 \cdot 2½H_2O$ (0.2326 g, 1.000 mmol) was dissolved in water (10 mL) followed by the addition of the same amount of nitric acid. The second solution was added dropwise with stirring to the hot acidified ligand mixture, which afforded dark-green solution. The latter was placed in a Petrie dish and allowed to evaporate. Well-shaped dark-green prismatic crystals that formed were picked by hand, dried, and used for the X-ray diffraction. (SI: Figure S1, Data S1).

FTIR (KBr, / cm^{-1})**:** 3583 (s, N–H), 3379 (br, O–H), 3000 (C–H), 1633 (s, C=N), 1596 (s, C=O), 1563 (s, C(=O)–N), 1454, 1435, 1381, 1347, 1220, 1160, 1113, 1067 (s, N–O), 1032 (s, N–O), 926, 841, 766, 739.

(2) = $[\{Cu(II)L3H_{-1}\}^{+}(OH_2)]_2^{2+} \cdot 2BF_4^{-} \cdot 2H_2O$

Synthesis S2.

To ligand L3 (0.0755 g, 0.2865 mmol), synthesised as previously reported (Nikolayenko, I.; Barry, J.; Chalmers, C. In Proceedings of the 37th International Conference on Coordination Chemistry, Cape Town, South Africa, 13–18 August 2006; p. 245), was added deionised water (7.5 mL) and the mixture heated with stirring. Aqueous $Cu(BF_4)_2$ (0.1000 M, 2,565 μL, 0.2565 mmol) was added. The resulting blue solution was boiled with stirring for 15 min, upon which the *p*H was adjusted to the desired value of 4 by dropwise addition of aqueous LiOH (0.5005 M) and HBF_4 (1.000 M). The solution of deep-blue colour, was filtered and mother liquor set aside for crystallisation. The dark-green prismatic crystals that formed were picked by hand, dried, and used for characterisation. (SI: Figure S2, Data S2).

FTIR (KBr, / cm^{-1})**:** 3570 (N–H), 3367 (s, O–H_w), 3255, 2956, 2871 (C–H), 1653 (s, C=N), 1620 (s, C=O), 1535 (s, C(=O)–N), 1453, 1444, 1381, 1343, 1202, 1160, 1125, 1084 (s, B–F), 1035 (s, N–O), 967, 844, 777, 683, 564, 523.

(3) = $[\{Cu(II)L3H_{-1}\}^{+}Cl^{-}]_2^{0} \cdot 4H_2O$

Synthesis S3.

A series of samples with the target volume between 5.0 mL and 5.5 mL were prepared in sealed glass vials from the following aqueous solutions: $[L3]_0$ = 4.999 mM in EtOH(abs), $[HCl]_0$ = 0.1987 M, $[CuCl_2]_0$ = 0.1942 M in HCl(aq, 1 mM), $[KOH]_0$ = 0.1036 M and one of the bulky counterion salts, $[TPPB]_0$ = 49.94 mM, $[PHFP]_0$ = 50.05 mM, $[PHFA]_0$ = 50.00 mM (where TPPB = tetraphenylphosphonium bromide, PHFP = potassium hexafluorophosphate, and PHFA = potassium hexafluoroarsenate). The target concentrations of the ingredients in the final solution were 1.0 mM for [L3], 1.0 mM or 2.0 mM for $[Cu^{2+}]$, 1.0 mM for [HCl], and 1.0 mM or 2.0 mM [counterion]. The balance of the volume was made up with deionised water and variable amount of aqueous potassium hydroxide to reach the desired *p*H value in the range 4.8 to 10.3. In a particular experiment, to 1000 μL of the ligand solution was added 3949 μL of water, followed by 25.5 μL of hydrochloric acid, 25.7 μL of aqueous copper(II) chloride and 40.81 μL of aqueous potassium hydroxide to reach the target *p*H value of 7.5. In this particular case, no solution of bulky counterion salt was added. The prepared mixture was stirred overnight and left for slow solvent evaporation (the vial was sealed with a plastic lid that had a pin-hole in it). After sufficient time a mixture of solid products precipitated out of solution. Well-formed dark-green prismatic crystals were picked by hand, dried, and subjected to further examination.

(4) = $[\{Cu(II)L3H_{-1}\}^{+}Cl^{-}]_2^{0} \cdot L3 \cdot 2H_2O$

Synthesis S4.

In a procedure similar to the above but with different quantities of water and potassium hydroxide (the target *p*H value of 8.6) complex (**4**) was isolated.

(5) = $[\{Li(OH_2)_3\}^{+}\{Cu(II)L2H_{-3}\}^{-}]^{0} \cdot H_2O$

Synthesis S5.

To ligand L2 (0.1218 g, 0.4887 mmol), synthesised as described in [1], was added aqueous LiOH (0.5005 M, 2×2,500 µL, 2.503 mmol) and the mixture stirred until dissolution. Aqueous $Cu(BF_4)_2$ (0.1000 M, 2×2,500 µL, 0.5000 mmol) was added, which caused instantaneous formation of wine-red solution. The latter was heated on a water bath, filtered, and the mother liquor allowed to evaporate. The resulting solid residue was re-dissolved in hot MeOH (20 mL), the remaining solid filtered off under vacuum, and the mother liquor left to evaporate. The needle-like red crystals that formed were picked by hand, dried, and used for characterisation. (SI: Figures S3-S4, Data S3-S6).

CHN: Calcd. for $C_9H_{21}N_4O_8CuLi$ (383.7727): C, 28.17; H, 5.52; N, 14.60. Found (UniFi): C, 28.70; H, 4.58; N, 14.12.

FTIR (KBr, / cm^{-1})**:** 3425 (br, O–H$_w$), 2924, 2856 (CH), 1598 (s, C=N), 1585 (s, C=O), 1394 (s, C(=O)–N), 1369, 1235, 1191, 1098 (s, N–O), 977, 746, 703, 530.

^{1}H NMR (DMSO-d$_6$, δ/ ppm)**:** NMR spectrum of this complex shows strong paramagnetic broadening, which prevented assignment of resonance signals.

MS-ToF [ES+] **m/z(%):** Calculated for $C_{18}H_{28}N_8O_8{}^{63}Cu_2Na \equiv [M_2Na]^+$ 633.0520; found 633.0533 (92); δ +2.1. Calculated for $C_{18}H_{28}N_8O_8{}^{63}Cu^{65}CuNa \equiv [MM'Na]^+$ 635.0500; found 633.0507 (81); δ +1.1. Calculated for $C_{18}H_{28}N_8O_8{}^{65}Cu_2Na \equiv [M'_2Na]^+$ 637.0482; found 637.0491 (23); δ +1.4.

Mass-spectroscopic data indicate that this complex is present in the gas phase as a mono-cationic dimer, where the charge is provided by the inclusion of a sodium cation.[2] Satisfactory composition was observed in the spectrum for ^{63}Cu and ^{65}Cu isotopes. It is worth noting that no such spectra were obtained for similar complexes with L1 and L3, where fragmentation of the complexes was observed. This serves as further confirmation of higher thermodynamic stability of the pseudomacrocyclic complex anion with propane link.

(6) = $[PPh_4]^{+} \cdot [Ni(II)L2H_{-3}]^{-} \cdot H_2O$

Synthesis S6.

To a hot solution of ligand L2 (0.5002 g, 2.007 mmol) in water (25 mL) was added with stirring a solution of $Ni(NO_3)_2{\cdot}6H_2O$ (0.6115 g, 2.103 mmol) in water (20 mL), which afforded a yellow solution with a green hue. Further addition of aqueous KOH (0.5 M × 12.6 mL, 6.3 mmol) deepened the yellow colour to amber. The mixture was left stirring at 80 °C for 1 hour. Tetraphenylphosphonium bromide, $[PPh_4]Br$, (0.8678 g, 2.008 mmol) was dissolved in a water-methanol (18 ml : 2 ml) mixture and added to the hot complex solution, which caused instantaneous precipitation of pale-yellow powder. The reaction mixture was cooled and left stirring overnight at room temperature. The mixture consisting of pale-yellow powdery precipitate and orange-yellow solution was brought to the boil and the solid phase removed by filtration. A bright-yellow powder (1.050 g), (**6**), was air-dried. The mother liquor was placed in a Petrie dish and allowed to evaporate. The well-shaped yellow prismatic crystals that formed were filtered off and left to dry in the air (0.195 g). These crystals were also identified as (**6**). XRD analysis revealed that the complex isolated had the same crystal structure as reported in [14].

Combined yield: 1.245 g, 97.0 %.

FTIR ($\bar{\nu}$ / cm^{-1})**:** 3442 (s, O–H$_w$), 3297, 2923, 2716, 2652 (C–H), 1692, 1647 (s, C=N), 1622 (s, C=O), 1585 (s, C=N), 1524, 1402 (s, C(=O)–N), 1352, 1176, 1107 (N–O), 958 (N–O), 832, 702, 661.

[2] Sodium cations are often responsible for the charge of molecular ions. Their source is the glassware used for handling the sample solution being nebulised by means of electrospray ionisation.

NMR: For this complex we have recorded ^{1}H, ^{13}C, dept135, ^{15}N, ^{31}P, COSY, HSQC, HMBC (^{1}H-^{13}C), and HMBC (^{1}H-^{15}N) spectra, SI: Figures S6-S16, which allowed the following assignment of chemical shifts.

Ni(II)-complex anion:

^{1}**H** (DMSO-*d*$_6$, δ/ ppm)**:** 1.335 (m, 2H, CH_2**CH_2**CH_2), 1.698 (s, 6H, CH_3), 2.641 (m, 4H, N**CH_2**), 18.816 (s, 1H, O···H–O).

^{13}**C** (DMSO-*d*$_6$, δ / ppm)**:** 10.77 (t, CH_3), 30.96 (t, CH_2**CH_2**CH_2), 40.31 (t, NCH_2), 149.51 (s, C(=NOH)), 169.03 (s, C(=O)).

^{15}**N** (DMSO-*d*$_6$, δ / ppm)**:** 108.59 (s, C(=O)–N), 303.19 (s, C(=NOH)).

Tetraphenylphosphonium cation:

^{1}**H** (DMSO-*d*$_6$, δ / ppm)**:** 7.713-7.753, 7.734-7.769 (m, 8H, H_A, $H_{A'}$), 7.792-7.784 (m, 8H, H_B, $H_{B'}$), 7.942-7.991 (m, 4H, H_C).

^{31}**P** (DMSO-*d*$_6$, δ / ppm): 22.32 (m,).

On the basis of experimental ^{1}H, ^{13}C, and HSQC spectra, an NMR spectral simulation was performed, which quantitatively reproduced the complex multiplets in the ^{1}H spectrum for the TPP cation, SI: Figure S17, and thus allowed to evaluate various nuclei correlations and the H-H, C-H, and P-C coupling constants for this ion, SI: Scheme S1, Table S3.

MS-ToF [ES+] **m/z:** Calculated for $C_{18}H_{29}N_8O_8{}^{58}Ni_2$ ≡ $[M_2H]^+$ 601.0815; found 601.0814; δ -0.2 ppm. Recording this signal required high cone voltage of 16.7 kV. The detected molecular ion was a monohydrogen cation of the neutral Ni(II) complex dimer of ligand L2. The isotopic composition in the mass-spectrum was as expected for nickel.

Besides (**6**), other anionic Ni(II)-complexes with ligand L2 and solvated Li^+, K^+ and Ni^{2+} in the role of counter-cation have been isolated. The NMR spectra of these compounds in DMSO-*d*$_6$ revealed virtual identity (within experimental errors) of the chemical shifts of the complex anion with those recorded for (**6**), which indicated high degree of stability of the complex anion in solution.

(7)–(8) = $[\mathrm{Ni(II)L2'H_{-2}}]^0$

Polymorphs (**7**) and (**8**) co-crystallised from the reaction mixture and the different crystals were picked by hand for the diffraction and other analyses.

Synthesis S7-S8.

Route A: To a solution of ligand L2 (1.2474 g, 5.00 mmol) in dry methanol (100 mL) was added a solution of $Ni(NO_3)_2 \cdot 6H_2O$ (1.5330 g, 5.57 mmol) in dry methanol (80 mL) and the obtained mixture left stirring for 30 min at room temperature, during which time the solution remained salad-green in colour. KOH (aq, 1.0 M×10 mL, 10 mmol) was added dropwise, which caused a rapid colour change from avocado-green to mustard, and the last 2.5 mL brought about ample precipitation. The resulting solution was brought to the boil and refluxed for 30 min. After about 15 min the colour changed to warm yellow, and by the end of reflux the precipitate has completely dissolved, leaving clear tea-coloured solution. H_2O_2 (aq[3], 2.554 mL, 25.00 mmol) was added with no visible change observed. The solution was left stirring in a stoppered flask for two months. The resulting mixture contained substantial amount of red and yellow precipitate in orange-red liquor. The primary precipitate was filtered off and dried on a watch glass to produce scarlet powder (0.404 g). Under the microscope one could see that it consisted of some unreacted L2, yellow complex (**6**), and new red nickel complex. This conclusion was confirmed by the ^{1}H NMR spectrum in DMSO-*d*$_6$, where the compounds were detected in approximately 0.37 : 0.25 : 0.39 ratio. The mother liquor was left in a beaker to evaporate, which afforded more of the precipitate. This secondary precipitate (0.860 g) consisted of bright-yellow powder, small yellow crystals, and pink-red crystals. The secondary mother liquor was also placed in a beaker and allowed to evaporate. This produced more yellow crystals and some thin red needle-like crystals. In summary, this route afforded considerable if incomplete conversion of YNC into RNC, (**7-8**). At this stage it is difficult to say whether the conversion ratio from yellow to red complex is a result of chemical equilibrium or a slow kinetics of oxidation. Indirect evidence indicates that the latter is more probable. A quantitative separation of RNC from YNC has not been carried out, but an obvious way of achieving this would be to exploit the

[3] ACS reagent, 30 wt. % in water.

difference in solubility of two complexes in mildly polar solvents, such as $CHCl_3$, EtOEt, EtOAc, or CH_2Cl_2 (YNC is a salt with anionic Ni-core, while RNC is a neutral complex). To the secondary precipitate was added water and the mixture brought to the boil with stirring. The remaining precipitate was filtered off and mother liquor extracted with ethyl acetate (60 mL). While the aqueous phase remained delicate yellow, the organic phase turned raspberry-pink. Separation and evaporation of the organic phase produced a solid containing two crystalline materials – well shaped yellow prisms and small clusters of red prisms. Combined yield: about 40 % (an estimate based on the relative intesity of NMR siganls in the reaction mixture).

Route B: To a honey-yellow solution of complex (**6**) (0.6342 g, 0.8991 mmol) in dry methanol (40 mL) was added H_2O_2 (aq, 511 μL, 5.00 mmol) with no visible change observed. The solution was left stirring in a stoppered flask for two months. The resulting red mixture was allowed to evaporate in a beaker, which produced a mixture of orange-yellow and wine-red crystals (0.487 g). The latter were fused and impossible to separate mechanically. The solid phase was washed with water (40 mL), the residual solid filtered off and allowed to dry in the air. Under the microscope it consisted of bright yellow powder, pale-yellow fused crystals and pale-red fused crystals. Another portion of water (50 mL) was added to the above solid (0.442 g) and the mixture heated with stirring. This afforded an orange solution and residual yellow precipitate. The former was extracted with ethyl acetate (50 mL). The organic phase, which contained the bulk of RNC was allowed to evaporate in a beaker. This produced long thin red crystal ribbons as well as small red prisms, small orange prisms, and very small yellow prisms. Analysis of the red material confirmed it as RNC. Another sample rich in RNC was treated with chloroform, the undissolved residual L2 and YNC filtered off, and the mother liquor left in a small beaker covered with a lid with a pinhole in it for slow evaporation. This produced nearly pure RNC, which crystallised as wonderfully long thin raspberry swords (*ca*. 0.2 mm × 0.2 mm × 25 mm). Besides chloroform, this complex was crystallised in a similar shape out of ethyl acetate, methanol, acetonitrile, and dimethyl sulfoxide. Combined yield: between 21 and 25 % (an estimate based on the NMR data).

For this novel complex we have recorded 1D (^{1}H, ^{13}C and ^{15}N) and 2D correlation (COSY, HSQC, HMBC (^{1}H-^{13}C), HMBC (^{1}H-^{15}N) spectra in both $CDCl_3$ and DMSO-d_6 solvents, which were sufficient for complete assignment of chemical shifts.

^{1}H NMR ($CDCl_3$/ ppm)**:** 2.04 (s, 6H, CH_3), 5.44 (t, 1H, *J* = 6.5 Hz, CHC**H**CH), 7.77 (d, 2H, *J* = 6.5 Hz, NC**H**), 18.24 (s, 1H, O···H–O).

^{13}C NMR ($CDCl_3$/ ppm)**:** 10.65 (q, CH3), 100.63 (t, CH**C**HCH), 148.27 (s, C(=N–O)), 152.67 (d, NCH), 171.64 (s, C(=O)).

^{1}H NMR (DMSO-d_6/ ppm)**:** 1.92 (s, 6H, CH_3), 5.57 (t, 1H, *J* = 6.2 Hz, CHC**H**CH), 7.75 (d, 2H, *J* = 6.2 Hz, NC**H**), 18.20 (s, 1H, O···H–O).

^{13}C NMR (DMSO-d_6/ ppm)**:** 10.84 (q, CH_3), 101.39 (t, CH**C**HCH), 148.47 (s, C(=N–O)), 153.33 (d, NCH), 171.57 (s, C(=O)).

MS-ToF [ES+] **m/z:** Calculated for $C_{18}H_{20}N_8O_8{}^{58}Ni_2Na$ ≡ $[M_2Na]^+$ 615.0009; found 615.0005; δ -0.7 ppm. Mass-spectroscopic data indicate that this complex is present in the gas phase as a monocationic dimer of the neutral Ni(II) complex of oxidised ligand L2′ with the charge provided by the inclusion of a sodium cation. Spectral lines corresponding to complexes of various Ni isotopes, namely, $^{58}Ni_2$, $^{58}Ni\,^{60}Ni$, $^{58}Ni\,^{61}Ni$, $^{58}Ni\,^{62}Ni$, $^{58}Ni\,^{64}Ni$, and $^{60}Ni_2$, were observed at intensities consistent with the abundance of these isotopes.

Structure Refinement

SR (1) = $[\{Cu(II)L1H_{-1}\}^+(OH_2)]_2^{2+} \cdot 2NO_3^- \cdot H_2O$

Hydrogen atom linked to the oxime group was localized in the Fourier difference map and freely refined. Hydrogen atoms linked to the coordinated water molecule were localized in the Fourier difference map and introduced in the calculation using the AFIX 7 instruction for regular geometry. Hydrogen atoms linked to the not coordinated water molecule were not localized in the Fourier difference map and not introduced in the calculation.

SR (2) = $[\{Cu(II)L3H_{-1}\}^+(OH_2)]_2^{2+} \cdot 2BF_4^- \cdot 2H_2O$

Hydrogen atoms linked to the oxime group and to the water molecules were localized in the Fourier difference map and freely refined. The anion is affected by rotational disorder; three of its fluorine atoms were found spread over two positions, each one treated with fixed 0.5 population parameter.

SR (3) = $[\{Cu(II)L3H_{-1}\}^{+}Cl^{-}]_2^0 \cdot 4H_2O$

Hydrogen atom linked to the oxime group was localized in the Fourier difference map and freely refined. Hydrogen atoms linked to the water molecules were localized in the Fourier difference map and introduced in the calculation using the AFIX 7 instruction for regular geometry.

SR (4) = $[\{Cu(II)L3H_{-1}\}^{+}Cl^{-}]_2^0 \cdot L3 \cdot 2H_2O$

Hydrogen atoms linked to the oxime group were localized in the Fourier difference map and introduced in the calculation. One of them was freely refined, while the other one (H1) was constrained to be 0.9 Å apart from the linked oxime oxygen (O1). Hydrogen atoms linked to the water molecule were localized in the Fourier difference map and introduced in the calculation using the AFIX 7 instruction for regular geometry.

SR (5) = $[\{Li(OH_2)_3\}^{+}\{Cu(II)L2H_{-3}\}^{-}]^0 \cdot H_2O$

Hydrogen atom linked to the oxime group was localized in the Fourier difference map and freely refined. Hydrogen atoms linked to the water molecules were localized in the Fourier difference map and introduced in the calculation using the AFIX 7 instruction for regular geometry.

SR (7) = $[Ni(II)L2'H_{-2}]^0$
The compound crystallizes in the acentric monoclinic space group Cc. The correct assignment of the space group, i.e. the lack of proper 2-fold axes and of the consequently generated inversion centres, was verified by careful inspection of the crystal packing.
Hydrogen atom linked to the oxime group was localized in the Fourier difference map and freely refined. Hydrogen atoms linked to the water molecules were localized in the Fourier difference map and introduced in the calculation using the AFIX 7 instruction for regular geometry.

SR (8) = $[Ni(II)L2'H_{-2}]^0$

Hydrogen atoms linked to the oxime group were localized in the Fourier difference map and freely refined.
